# Association between triglyceride-glucose related indices with the all-cause and cause-specific mortality among the population with metabolic syndrome

**DOI:** 10.1186/s12933-024-02215-0

**Published:** 2024-04-24

**Authors:** Xiaoyuan Wei, Yu Min, Ge Song, Xin Ye, Lei Liu

**Affiliations:** 1 Department of Head and Neck Oncology, West China Hospital, Sichuan University, Chengdu, Sichuan 610041 P.R. China; 2 Department of Biotherapy, West China Hospital, Sichuan University, Chengdu, 610041 P.R. China; 3 Department of Oncology, Chengdu University of Traditional Chinese Medicine, Chengdu, 610041 P.R. China

**Keywords:** Metabolic syndrome, TyG index, TyG-WC index, TyG-WHtR index, All-cause mortality, Cardiovascular mortality, Diabetes mortality

## Abstract

**Background:**

Triglyceride-glucose (TyG) index has been determined to play a role in the onset of metabolic syndrome (MetS). Whether the TyG index and TyG with the combination of obesity indicators are associated with the clinical outcomes of the MetS population remains unknown.

**Method:**

Participants were extracted from multiple cycles of the National Health and Nutrition Examination Survey (NHANES) between 1999 and 2018 years. Three indicators were constructed including TyG index, TyG combining with waist circumference (TyG-WC), and TyG combining with waist-to-height ratio (TyG-WHtR). The MetS was defined according to the National Cholesterol Education Program (NCPE) Adult Treatment Panel III. Kaplan-Meier (KM) curves, restricted cubic splines (RCS), and the Cox proportional hazard model were used to evaluate the associations between TyG-related indices and mortality of the MetS population. The sensitive analyses were performed to check the robustness of the main findings.

**Results:**

There were 10,734 participants with MetS included in this study, with 5,570 females and 5,164 males. The median age of the study population was 59 years old. The multivariate Cox regression analyses showed high levels of TyG-related indices were significantly associated with the all-cause mortality of MetS population [TyG index: _adjusted_hazard ratio (aHR): 1.36, 95%confidence interval (CI): 1.18–1.56, *p* < 0.001; TyG-WHtR index: aHR = 1.29, 95%CI: 1.13–1.47, *p* < 0.001]. Meanwhile, the TyG-WC and TyG-WHtR index were associated with cardiovascular mortality of the MetS population (TyG-WC: aHR = 1.45, 95%CI: 1.13–1.85, *p* = 0.004; TyG-WHtR: aHR = 1.50 95%CI: 1.17–1.92, *p* = 0.002). Three TyG-related indices showed consistent significant correlations with diabetes mortality (TyG: aHR = 4.06, 95%CI: 2.81–5.87, *p* < 0.001; TyG-WC: aHR = 2.55, 95%CI: 1.82–3.58, *p* < 0.001; TyG-WHtR: aHR = 2.53 95%CI: 1.81–3.54, *p* < 0.001). The RCS curves showed a non-linear trend between TyG and TyG-WC indices with all-cause mortality (p for nonlinearity = 0.004 and 0.001, respectively). The sensitive analyses supported the positive correlations between TyG-related indices with mortality of the MetS population.

**Conclusion:**

Our study highlights the clinical value of TyG-related indices in predicting the survival of the MetS population. TyG-related indices would be the surrogate biomarkers for the follow-up of the MetS population.

**Supplementary Information:**

The online version contains supplementary material available at 10.1186/s12933-024-02215-0.

## Introduction

Metabolic syndrome (MetS), defined by a cluster of metabolic dysfunctions, is considered to be a major global public health problem, given the increasingly high prevalence in the general population worldwide [1–3]. Among the U.S. population, it was estimated that over 30% of the population had MetS, especially in terms of older adults [4, 5]. Most recently, the epidemiological study based on the U.S. population revealed that the overall trend of MetS incidence has increased from 27.6 to 32.3% during past decades [6]. Accumulating evidence suggested that the series of symptoms in MetS conditions such as insulin resistance, high blood pressure, and obesity would not only increase the short- and long-term risks for chronic metabolic diseases but also cause premature death in later life [6–9]. Nevertheless, identifying the prognostic biomarkers and designing tailored follow-up advice for the MetS population remains challenging [10].

Notably, the triglyceride-glucose (TyG) index has been determined to be a simple tool for detecting insulin resistance and evaluating cardiovascular disease risks among the general population [11]. It showed promising value in predicting the onset and progress of multiple metabolic diseases [12]. Consistently, emerging evidence highlighted that the TyG index also maintained the high capability of identifying individuals at high risk of developing MetS [13–18]. For example, one large-scale longitudinal study conducted in Korea showed that the TyG index had a high predictive value in determining the long-term risk for developing MetS among the Korean population [16]. Similar findings were also observed in other countries with varied ethnicity [13, 18, 19]. Recently, robust evidence derived from the meta-analysis revealed that the pool sensitivity and specificity of the TyG index for MetS screening were all above 80%, which validates the clinical utility of this index [19]. On the other hand, the TyG index was recently identified to be associated with the prognosis of the population with comorbidities, especially in critically ill groups [20–22]. However, limited studies have been conducted to further investigate the predictive role of the TyG index in the prognosis of the MetS population. The positive association between the TyG index and mortality of the MetS population was only observed in subgroup patients with comorbidities [23]. Whether it would be a surrogate biomarker for health care in the MetS population, especially in countries with high MetS burdens, remains unknown. Meanwhile, the latest evidence suggested that the TyG index with the combinations of adiposity indicators, including but not limited to TyG-waist circumference (TyG-WC) and waist-to-height ratio (TyG-WHtR), showed higher predictive performance than the single TyG index in predicting the survivals of patients [24–26]. However, the association between these derived indices with the survival of MetS among the American population is less studied.

Therefore, we aim to explore the association between the TyG-related indices with the all-cause as well as cause-specific mortality of the MetS population, based on a large-scale, prospective, population-based cohort in the U.S. This is the first epidemiological study that focuses on the prognosis links between the series of TyG-related indices and the MetS.

## Materials and methods

### Data source

The datasets were derived from ten cycles of interviews (1999 to 2018 years) in the National Health and Nutrition Examination Survey (NHANES) database. The NHANES database systematically gathered nationally representative health-related data on the repetitive non-institutionalized U.S. population, utilizing a stratified, multistage probability sampling design [27]. The specific descriptions of the NHANES database can be found on the website (https://www.cdc.gov/nchs/nhanes/).

### Population selection

**Fig. 1:**
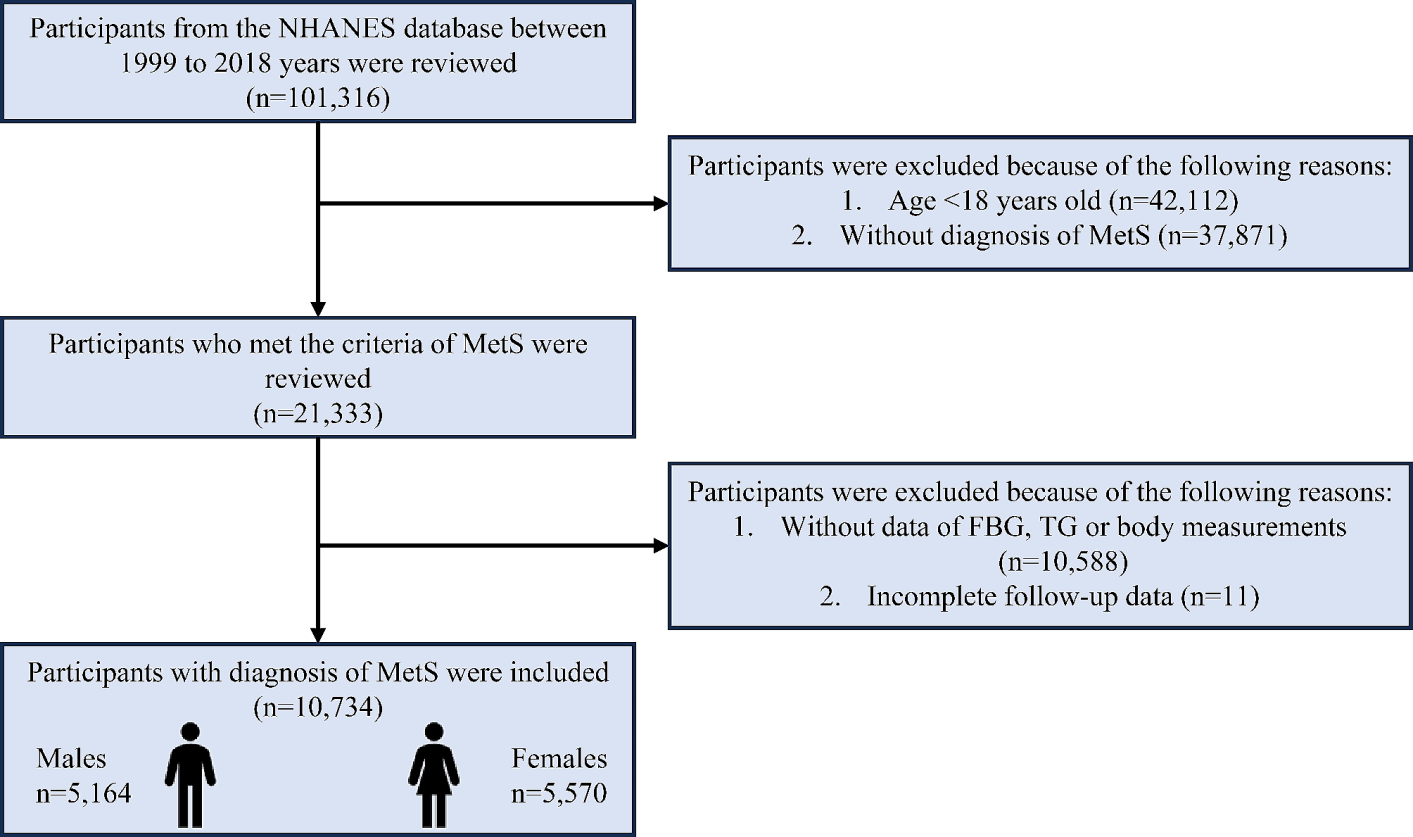
The participants’ selection process in the present study. A total of 101,316 participants from ten cycles of interviews between 1999 and 2018 years were reviewed and 10,734 participants with MetS were ultimately included. NHANES: National Health and Nutrition Examination Survey; MetS: metabolic syndrome; FBG: fasting blood glucose; TG: triglyceride

To evaluate the association between the TyG-related indices with the all-cause and cause-specific mortality of the MetS population, only participants with the diagnosis of MetS were included. Therefore, during the ten cycles of interviews between 1999 and 2018 years, there were 101,316 participants were reviewed. After excluding the participants aged below 18 years old or non-MetS, there were 21,333 participants were initially included. Additionally, participants without records of triglyceride (TG) and fasting blood glucose (FBG), body measurements, or loss to follow-up were further excluded. Ultimately, there were 10,734 participants were included in this study (Fig. 1).

### Definition of mets

The definition of MetS was based on the previous guideline [National Cholesterol Education Program (NCEP): National Cholesterol Education Program Adult Treatment Panel III] [28]. Participants who met three or more of the following criteria: [1] the FBG > 100 mg/dL or drug treatment for diabetes mellitus; [2] high-density lipoprotein cholesterol (HDL-C) < 50 mg/dL in females, < 40 mg/dL in males or drug treatment for reduced HDL-C; [3] Plasma TG > 150 mg/dL or drug treatment for raised TG; [4] Waist circumference > 88 cm in women or > 102 cm in men; [5] Blood pressure > 130/85 mmHg or drug treatment for raised blood pressure.

### TyG-related indices measurements

There were three TyG-related indices of interest measured in the present study. The TyG index was measured by the peripheral blood test (TG, and FBG) of the participants at baseline according to the previous literature [29, 30]. Two variables, including WC and WHtR, were selected as the obesity indicators in the MetS population [31]. Specifically, the indices of TyG, TyG-WC, and TyG-WHtR were calculated as the following equations [32–34]:

1 $${\rm{TyG}}\,{\rm{ = }}\,{\rm{ln}}\,\left[ {{\rm{TG}}\,\left( {{\rm{mg/dL}}} \right)\,{\rm{ \times }}\,{\rm{FBG}}\,\left( {{\rm{mg/dL}}} \right){\rm{/2}}} \right]$$

2 $$\eqalign{{\rm{TyG - WC}}\,{\rm{ = }}\, & {\rm{ln}}\,\left[ {{\rm{TG}}\,\left( {{\rm{mg/dL}}} \right)\,{\rm{ \times }}\,{\rm{FBG}}\,\left( {{\rm{mg/dL}}} \right){\rm{/2}}} \right]\, \cr & {\rm{ \times }}\,{\rm{waist}}\,{\rm{circumference}}\,\left( {{\rm{cm}}} \right) \cr}$$

3 $$\eqalign{{\rm{TyG - WHtR}}\,{\rm{ = }}\, & {\rm{ln}}\,\left[ {{\rm{TG}}\,\left( {{\rm{mg/dL}}} \right)\,{\rm{ \times }}\,{\rm{FBG}}\,\left( {{\rm{mg/dL}}} \right){\rm{/2}}} \right]{\rm{ }} \cr & {\rm{ \times waist}}\,{\rm{circumference}}\,\left( {{\rm{cm}}} \right){\rm{/height}}\,\left( {{\rm{cm}}} \right) \cr}$$

The MetS population was classified into four groups by the quartiles of the TyG index, TyG-WC index, and TyG-WHtR index, respectively, and the group at the 1st quartile was set as the reference.

### Covariates of interest

We collected the demographic information of participants from the NHANES database. Detailly, the socioeconomic characteristics included gender (male or female), age, race (Mexican, Hispanic, non-Hispanic White, non-Hispanic Black, or other races), marital status (not married, married or living with a partner), educational level (≤ high school, college, or > college), and family income-poverty ratio (< 1.3, 1.3–3.5, or > 3.5) were collected. Besides, the living habits and history of comorbidities include smoking status (never, ever, or current), alcohol use (never, ever, or current), history of cancer, chronic kidney disease (CKD), and cardiovascular disease (CVD) (yes or no) were further collected. In addition, physical and laboratory examinations such as body mass index (BMI), energy intake (average kilocalorie derived from two 24-hour dietary recall interviews), serum levels of estimated glomerular filtration rate (eGFR), blood urea nitrogen (BUN), total cholesterol (TC), high-density lipoprotein cholesterol (HDL-C), low-density lipoprotein cholesterol (LDL-C), uric acid (UA), glutamic-pyruvic transaminase (ALT), aspartate transaminase (AST), albumin (ALB), and total bilirubin (TBil) were also selected as the potential confounders.

### Outcome measurements

The main outcome of the current study was the all-cause mortality of the MetS population. The second outcome was the cause-specific mortality (cardiovascular and diabetes mortality) of the MetS population. The mortality data for the follow-up population were obtained from the NHANES public-use linked mortality file as of December 31, 2019, which was correlated with NCHS with the National Death Index (NDI) through a probability matching algorithm. The ICD-10 (International Statistical Classification of Diseases, 10th revision) was used to check the causes of mortality. The period of follow-up was calculated from the date when the interview was initially taken to either the date of the patient’s death or December 31, 2019 [35].

### Statistical analysis

Kolmogorov-Smirnov test was used to check the normality assumption distribution of each variable. The normally distributed variables were presented as mean (standard deviation). The non-normally distributed variables were presented as median (interquartile range). The categorical variables were presented as numbers (percentage, %). Continuous variables with normal distribution were evaluated using Student’s t-test, while continuous variables with non-normal distribution were tested using the Kruskal-Wallis test. Categorical variables were compared by using the Chi-Squared test. Univariate and multivariable Cox proportional hazard models were used to estimate the association of the TyG index with all-cause, cardiovascular as well as diabetic mortality of the MetS population.

The selections of controlled covariates in the present study were based on previous literature evaluating the survival of MetS [3, 8, 36]. As the WC, WHtR, and BMI were highly correlated, BMI was not adjusted during the Cox regression analysis for evaluating the association between TyG-WC and TyG-WHtR indices with the survival of the MetS population. Detailly, model 1 served as the unadjusted analysis. Besides, brief adjustments for age, gender, and race were made in Model 2. In the fully adjusted model, we accounted for age, gender, race, marital status, educational level, family income-poverty ratio, smoking status, alcohol use, cancer, CKD, CVD, BMI (only for TyG index), energy intake, serum levels of TC, HDL-C, LDL-C, BUN, UA, eGFR, ALT, AST, ALB, and TBil.

Kaplan-Meier (KM) curves were used to show the censored data and different survival patterns among the MetS population with different quartiles of the TyG index. Missing data (< 5%) was imputed by using the multiple imputation method. To evaluate the dose-effect correlations between TyG-related indices with all-cause and cause-specific mortality of the MetS population, restricted cubic splines (RCS) were employed. The selection of knots for the RCS curves was guided by the minimization of Akaike’s Information Criterion (AIC). The RCS curves were fitted using different numbers and positions of knots, and the corresponding AIC values were calculated for each model. The final knot configuration was chosen based on the model with the lowest AIC value, indicating the optimal balance between model fit and complexity. To check the robust connections between TyG-related indices and mortality of the MetS population, four sets of sensitive analyses were conducted to validate the main findings. First, we excluded participants who reported a history of diabetes mellitus at baseline. Second, we excluded participants who died within two years after the interview. Third, participants with a history of comorbidities were excluded. Last, participants with low or normal BMI were excluded.

All statistical analyses of the present study were conducted by using the R software (version 4.2.3, https://www.r-project.org/). To determine a more rigorous interpretation of the associations between TyG, TyG-WC, and TyG-WHtR with the survival of the MetS population, the Bonferroni-corrected P-value was used in this study [37]. Thus, the significance level *P* = 0.05 was divided by three, which provided a significance level corrected for multiple testing: *P* = 0.017.

## Results

### Demographic characteristics of the MetS population

During the study years, there were 10,734 participants with a diagnosis of MetS included in the present study (Table 1). The median age of the MetS population was 59 years and females counted for a slightly higher proportion than males (5,570 cases vs. 5,164 cases). However, males with Mets showed a higher mortality risk than females (1,182 cases vs. 1,033 cases). Nearly half of the MetS population were non-Hispanic white (4,940 cases) and less than 40% of them had a degree of college or above. More than 30% of the MetS population was overweight (3,573 cases) and nearly 57% were obese (6,051 cases). At the time of the interview, over half of the study population were still drinking (5,982 cases) and nearly 20% of them were still smoking (2,015 cases). The prevalence of comorbidities was 12.19% (1,309 cases) for cancer, 20.48% (2,198 cases) for CKD, and 14.48% (1,554 cases) for CVD, respectively. The median level of the TyG index was 7.41 and the TyG-WC as well as TyG-WHtR indices were 787.28 and 4.72, respectively. With a median follow-up of 100 months, 2,215 deaths were observed, with 360 for diabetes mortality and 615 for cardiovascular mortality. Non-survivors were observed to have characteristics of males, Non-Hispanic White, unmarried status, older age, less daily energy intake, lower educational level, lower BMI, higher levels of FBG, TyG index, and having a history of comorbidities. The detailed information of each group of the MetS population with varied quartile levels of TyG-related indices was summarized in Table S1-S3.

**Table 1:** The demographic characteristics of the MetS population in the present study were stratified by survival status

Variable	Total (*n* = 10,734)	Survivor (*n* = 8,519)	Non-survivors (*n* = 2,215)	P^*^
Age, M (Q_1_, Q_3_)	59.00 (45.00, 70.00)	55.00 (42.00, 65.00)	72.00 (63.00, 80.00)	**< 0.001**
Energy intake, M (Q_1_, Q_3_)	1848.00 (1384.25, 2423.00)	1865.00 (1438.50, 2495.00)	1665.00 (1237.00, 2152.50)	**< 0.001**
FBG, M (Q_1_, Q_3_)	108.00 (100.50, 124.00)	107.00 (100.00, 121.00)	112.40 (102.00, 138.00)	**< 0.001**
TyG, M (Q_1_, Q_3_)	7.41 (7.01, 7.80)	7.40 (7.00, 7.78)	7.45 (7.06, 7.88)	**< 0.001**
TyG-WC, M (Q_1_, Q_3_)	787.28 (711.75, 876.29)	786.40 (711.65, 876.61)	790.52 (712.16, 874.47)	0.596
TyG-WHtR, M (Q_1_, Q_3_)	4.72 (4.28, 5.25)	4.71 (4.27, 5.25)	4.76 (4.31, 5.26)	0.049
TC, M (Q_1_, Q_3_)	5.04 (4.32, 5.84)	5.07 (4.34, 5.84)	4.96 (4.21, 5.82)	**0.001**
TG, M (Q_1_, Q_3_)	1.65 (1.12, 2.29)	1.65 (1.12, 2.28)	1.64 (1.13, 2.30)	0.645
HDL-C, M (Q_1_, Q_3_)	45.00 (39.00, 54.00)	45.00 (39.00, 54.00)	45.00 (40.00, 55.00)	0.034
LDL-C, M (Q_1_, Q_3_)	2.97 (2.38, 3.58)	2.97 (2.43, 3.62)	2.97 (2.30, 3.45)	**< 0.001**
BUN, M (Q_1_, Q_3_)	4.64 (3.93, 6.07)	4.64 (3.57, 5.71)	5.71 (4.28, 7.50)	**< 0.001**
UA, M (Q_1_, Q_3_)	345.00 (285.50, 404.50)	339.00 (285.50, 398.50)	356.90 (297.40, 428.30)	**< 0.001**
eGFR, M (Q_1_, Q_3_)	87.10 (64.89, 113.80)	91.80 (70.20, 118.27)	66.36 (46.29, 91.56)	**< 0.001**
ALT, M (Q_1_, Q_3_)	22.00 (17.00, 30.75)	23.00 (17.00, 32.00)	20.00 (16.00, 26.00)	**< 0.001**
AST, M (Q_1_, Q_3_)	23.00 (19.00, 28.00)	23.00 (19.00, 28.00)	23.00 (19.00, 28.00)	0.816
ALB, M (Q_1_, Q_3_)	42.00 (39.00, 44.00)	42.00 (40.00, 44.00)	41.00 (39.00, 43.50)	**< 0.001**
TBil, M (Q_1_, Q_3_)	10.26 (8.55, 13.68)	10.26 (8.55, 13.68)	11.97 (8.60, 15.39)	**< 0.001**
Gender, n (%)				**< 0.001**
Female	5,570 (51.89)	4,537 (53.26)	1,033 (46.64)	
Male	5,164 (48.11)	3,982 (46.74)	1,182 (53.36)	
Race, n (%)				**< 0.001**
Mexican	1,966 (18.32)	1,646 (19.32)	320 (14.45)	
Hispanics	961 (8.95)	856 (10.05)	105 (4.74)	
Non-Hispanic White	4,940 (46.02)	3,608 (42.35)	1,332 (60.14)	
Non-Hispanic Black	2,067 (19.26)	1,682 (19.74)	385 (17.38)	
Others	800 (7.45)	727 (8.53)	73 (3.30)	
Education level, n (%)				**< 0.001**
≤High school	6,005 (55.94)	4,538 (53.27)	1,467 (66.23)	
College	2,904 (27.05)	2,439 (28.63)	465 (20.99)	
> College	1,825 (17.00)	1,542 (18.10)	283 (12.78)	
Marital status, n (%)				**< 0.001**
Not married	4,245 (39.55)	3,186 (37.40)	1,059 (47.81)	
Married or living with a partner	6,489 (60.45)	5,333 (62.60)	1,156 (52.19)	
Family income of poverty ratio, n (%)				**< 0.001**
<1.3	3,145 (29.3)	2,428 (28.50)	717 (32.37)	
1.3–3.5	4,821 (44.91)	3,720 (43.67)	1,101 (49.71)	
>3.5	2,768 (25.79)	2,371 (27.83)	397 (17.92)	
BMI, n (%)				**< 0.001**
<25	1,110 (10.34)	757 (8.89)	353 (15.94)	
≥25 and < 30	3,573 (33.29)	2,752 (32.30)	821 (37.07)	
≥30	6,051 (56.37)	5,010 (58.81)	1,041 (47.00)	
Smoking status, n (%)				**< 0.001**
Never	5,417 (50.47)	4,526 (53.13)	891 (40.23)	
Current	2,015 (18.77)	1,609 (18.89)	406 (18.33)	
Ever	3,302 (30.76)	2,384 (27.98)	918 (41.44)	
Alcohol use, n (%)				**< 0.001**
Never	2,453 (22.85)	1,702 (19.98)	751 (33.91)	
Current	5,982 (55.73)	5,011 (58.82)	971 (43.84)	
Ever	2,299 (21.42)	1,806 (21.20)	493 (22.26)	
Cancer, n (%)				**< 0.001**
No	9,425 (87.81)	7,663 (89.95)	1,762 (79.55)	
Yes	1,309 (12.19)	856 (10.05)	453 (20.45)	
CKD, n (%)				**< 0.001**
No	8,536 (79.52)	7,255 (85.16)	1,281 (57.83)	
Yes	2,198 (20.48)	1,264 (14.84)	934 (42.17)	
CVD, n (%)				**< 0.001**
No	9,180 (85.52)	7,637 (89.65)	1,543 (69.66)	
Yes	1,554 (14.48)	882 (10.35)	672 (30.34)	

^*^P-value < 0.017 was considered significant, as we had to correct our analysis for multiple testing (P-value of 0.017 was calculated as: 0.05 divided by 3)

Bold value means statistically significant

*Abbreviation* MetS: metabolic syndrome; M: median; Q1: 1st quartile; Q3: 3rd quartile; n: number; FBG: fasting blood glucose; TyG: triglyceride-glucose; TyG-WC: TyG combining with waist circumference; TyG-WHtR: TyG combining with waist-to-height ratio; TC: total cholesterol; TG: triglyceride; CKD: chronic kidney disease; CVD: cardiovascular disease; eGFR: estimated glomerular filtration rate; BUN: blood urea nitrogen; HDL-C: high-density lipoprotein cholesterol; LDL-C: low-density lipoprotein cholesterol; UA: uric acid; ALT: glutamic-pyruvic transaminase; AST: aspartate transaminase; ALB: albumin; TBil: total bilirubin; BMI: body mass index

### Survival patterns of MetS population in different quartile levels of TyG-related indices

**Fig. 2:**
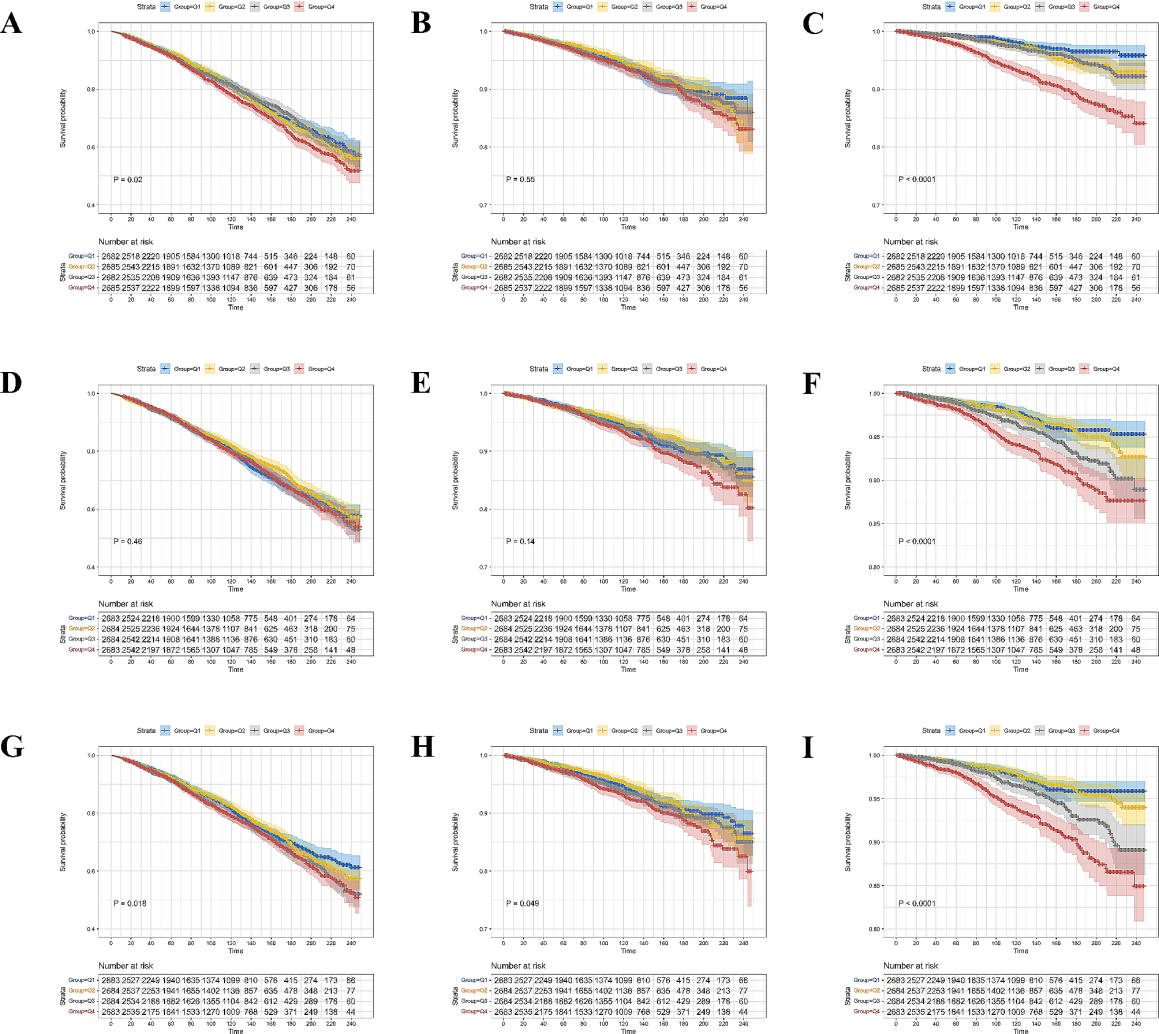
Kaplan-Meier curves show the overall and cause-specific survival probabilities of the MetS population with different quartile levels of TyG, TyG-WC, and TyG-WHtR indices. (**A**) overall survival with different quartiles of the TyG index; (**B**) cardiovascular-specific survival with different quartiles of the TyG index; (**C**) diabetes-specific survival with different quartiles of the TyG index; (**D**) overall survival with different quartiles of the TyG-WC index; (**E**) cardiovascular-specific survival with different quartiles of TyG-WC index; (**F**) diabetes-specific survival with different quartiles of TyG-WC index; (**G**) overall survival with different quartiles of TyG-WHtR index; (**H**) cardiovascular-specific survival with different quartiles of TyG-WHtR index; (**I**) diabetes-specific survival with different quartiles of TyG-WHtR index. MetS: metabolic syndrome; TyG: triglyceride-glucose; WC: waist circumference; WHtR: waist-to-height ratio

The KM curves showed that the MetS population at the low quartile levels of the TyG index group showed significantly higher overall as well as diabetes-specific survival probabilities when compared with those in the high quartile groups (*p* = 0.02 and *p* < 0.0001, respectively) (Fig. 2A and C). Additionally, significant differences were only observed in the diabetes-specific survival of the MetS population with varied quartiles of the TyG-WC index (*p* < 0.0001) (Fig. 2D-F). The TyG-WHtR index was adversely associated with overall and cause-specific survival among the MetS population (*p* = 0.018 for overall survival, *p* = 0.049 for cardiovascular-specific survival, and *p* < 0.0001 for diabetes-specific survival, respectively) (Fig. 2G and I).

### Association between TyG, TyG-WC, and TyG-WHtR indices with all-cause mortality of MetS population

**Fig. 3:**
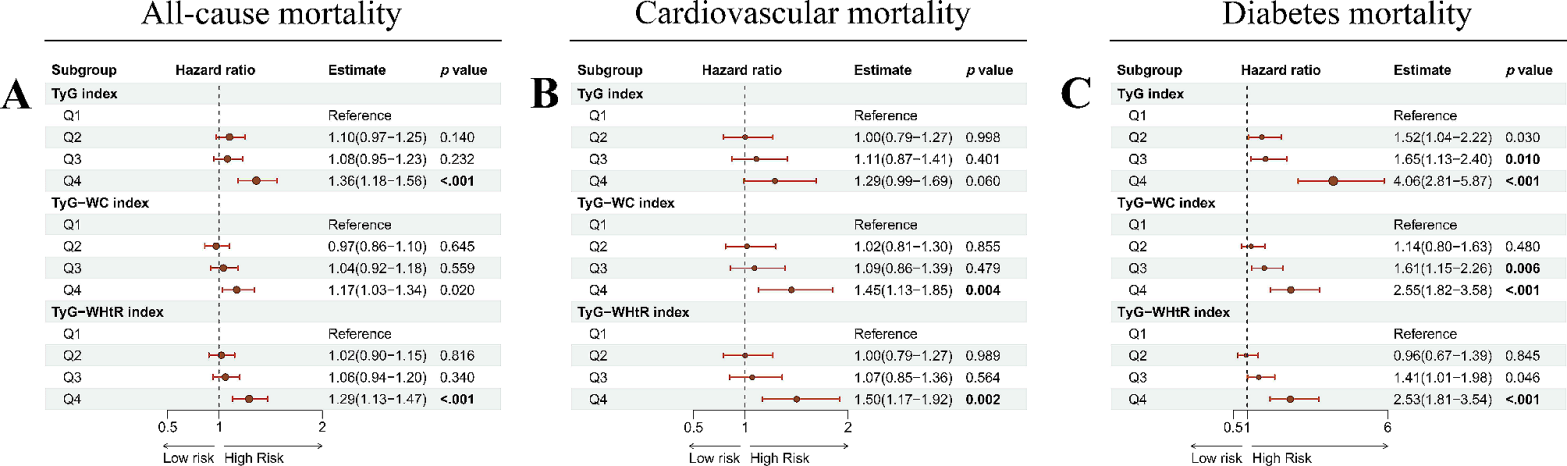
The forest plots show the associations between TyG, TyG-WC, and TyG-WHtR indices with all-cause and cause-specific mortality of the MetS population. B(**A**) the association between TyG, TyG-WC, and TyG-WHtR indices with all-cause mortality; (**B**) the association between TyG, TyG-WC, and TyG-WHtR indices with cardiovascular mortality; (**C**) the association between TyG, TyG-WC, and TyG-WHtR indices with diabetes mortality. The results were adjusted for age, gender, race, marital status, educational level, family income-poverty ratio, smoking status, alcohol use, cancer, CKD, CVD, BMI (only for TyG index), energy intake, serum levels of TC, HDL-C, LDL-C, BUN, UA, eGFR, ALT, AST, ALB, and TBil. A P-value < 0.017 was considered significant, as we had to correct our analysis for multiple testing (P-value of 0.017 was calculated as: 0.05 divided by 3). TyG: triglyceride-glucose; WC: waist circumference; WHtR: waist-to-height ratio; TC: total cholesterol; CKD: chronic kidney disease; CVD: cardiovascular disease; eGFR: estimated glomerular filtration rate; BUN: blood urea nitrogen; HDL-C: high-density lipoprotein cholesterol; LDL-C: low-density lipoprotein cholesterol; UA: uric acid; ALT: glutamic-pyruvic transaminase; AST: aspartate transaminase; ALB: albumin; TBil: total bilirubin; BMI: body mass index

The Cox regression analyses showed the association between TyG, TyG-WC, and TyG-WHtR indices with the all-cause mortality of MetS population [TyG index at 4th quartile: Unadjusted hazard ratio (HR) = 1.17, 95% confidence interval (CI): 1.04–1.31, *p* = 0.011; Less adjusted HR = 1.45, 95%CI: 1.28–1.63; *p* < 0.001; Full adjusted HR = 1.36, 95%CI: 1.18–1.56, *p* < 0.001 (**Table S4**); TyG-WC index at 4th quartile: Unadjusted HR = 1.01, 95% CI: 0.90–1.14, *p* = 0.868; Less adjusted HR = 1.40, 95%CI: 1.24–1.58; *p* < 0.001; Full adjusted HR = 1.17, 95%CI: 1.03–1.34, *p* = 0.020 (**Table S5**); TyG-WHtR index: Unadjusted HR = 1.16, 95% CI: 1.03–1.30, *p* = 0.017; Less adjusted HR = 1.54, 95%CI: 1.37–1.74; *p* < 0.001; Full adjusted HR = 1.29, 95%CI: 1.13–1.47, *p* < 0.001 (**Table S6**)] (Fig. 3A).

### Association between TyG, TyG-WC, and TyG-WHtR indices with cause-specific mortality of MetS population

In our analyses, both of the TyG-WC and TyG-WHtR indices were significantly associated with the cardiovascular mortality of the MetS population [TyG-WC index: Unadjusted HR = 1.21, 95% CI: 0.97–1.51, *p* = 0.090; Less adjusted HR = 1.73, 95%CI: 1.37–2.17; *p* < 0.001; Full adjusted HR = 1.45, 95%CI: 1.13–1.85, *p* = 0.004 (**Table S5**); TyG- WHtR index: Unadjusted HR = 1.29, 95% CI: 1.03–1.61, *p* = 0.026; Less adjusted HR = 1.83, 95%CI: 1.46–2.30 *p* < 0.001; Full adjusted HR = 1.50, 95%CI: 1.17–1.92, *p* = 0.002 (**Table S6**)] (Fig. 3B). However, a borderline positive correlation was observed between the TyG index and cardiovascular mortality with quartile classification [(Full adjusted: HR = 1.29, 95%CI: 0.99–1.69, *p* = 0.060 (**Table S4**)] (Fig. 3B).

Meanwhile, the three TyG-related indices were all significantly association with diabetes mortality [TyG index: Full adjusted HR = 4.06, 95%CI: 2.81–5.87, *p* < 0.001(**Table S4**); TyG-WC index: Full adjusted HR = 2.55, 95%CI: 1.82–3.58, *p* < 0.001 (**Table S5**); TyG-WHtR index: Full adjusted HR = 2.53, 95%CI: 1.81–3.54, *p* < 0.001 (**Table S6**)] (Fig. 3C).

### Non-linear trends of TyG-related indices with mortality of MetS population

**Fig. 4:**
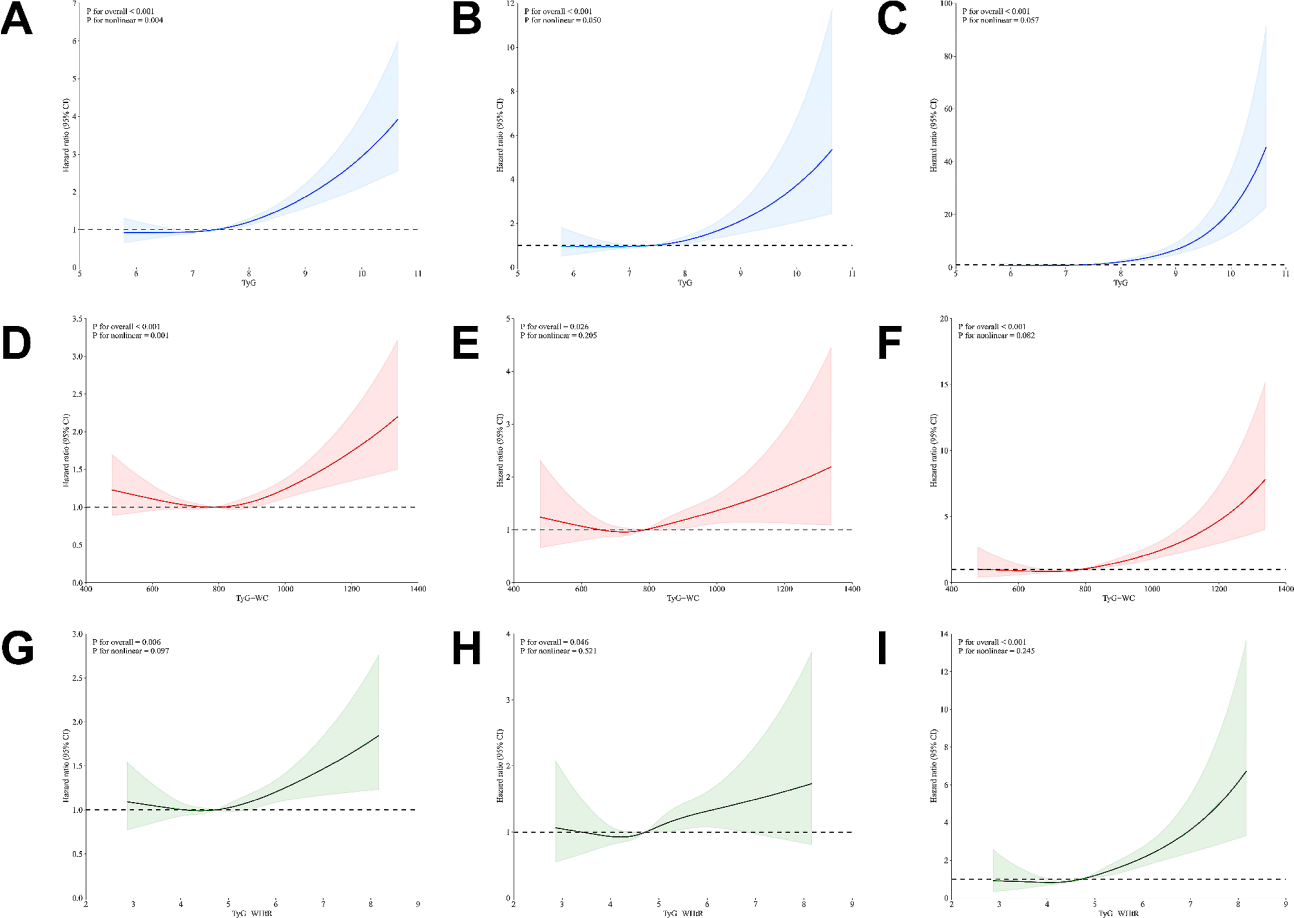
Restricted cubic spline of the linear trends between TyG, TyG-WC, and TyG-WHtR indices with all-cause and cause-specific mortality of MetS population. (**A**) all-cause mortality risk with different levels of the TyG index; (**B**) cardiovascular mortality risk with different levels of the TyG index; (**C**) diabetes mortality risk with different levels of the TyG index; (**D**) all-cause mortality risk with different levels of TyG-WC index; (**E**) cardiovascular mortality risk with the different level of TyG-WC index; (**F**) diabetes mortality risk with the different level of TyG-WC index; (**G**) all-cause mortality with the different level of TyG-WHtR index; (**H**) cardiovascular mortality risk with the different level of TyG-WHtR index; (**I**) diabetes mortality risk with the different level of TyG-WHtR index. The dotted lines represent 95% confidence intervals. Spline analyses were adjusted for age, gender, race, marital status, educational level, family income-poverty ratio, smoking status, alcohol use, cancer, CKD, CVD, BMI (only for TyG index), energy intake, serum levels of TC, HDL-C, LDL-C, BUN, UA, eGFR, ALT, AST, ALB, and TBil. TyG: triglyceride-glucose; WC: waist circumference; WHtR: waist-to-height ratio; TC: total cholesterol; CKD: chronic kidney disease; CVD: cardiovascular disease; eGFR: estimated glomerular filtration rate; BUN: blood urea nitrogen; HDL-C: high-density lipoprotein cholesterol; LDL-C: low-density lipoprotein cholesterol; UA: uric acid; ALT: glutamic-pyruvic transaminase; AST: aspartate transaminase; ALB: albumin; TBil: total bilirubin; BMI: body mass index

As shown in Fig. 4, the multivariable-adjusted RCS analyses were conducted to visualize the relation of TyG-related indices with all-cause and cause-specific mortality in the MetS population. Specifically, a Non-linear trend was observed in the relationships between TyG as well as TyG-WC indices with the all-cause mortality (P for non-linearity = 0.004 and 0.001, respectively) (Fig. 4A and D). However, the TyG-WHtR index showed a linear association with the all-cause mortality of the MetS population (P for overall = 0.006) (Fig. 4G). The RCS curves showed broad line significant non-linear associations between the TyG index and cause-specific mortality (P for non-linearity = 0.050 and 0.057, respectively) (Fig. 4B and C). Besides, TyG-WC and TyG-WHtR indices presented consistent linear associations with cardiovascular and diabetes mortality (all P for overall < 0.05) (Fig. 4E, F, H and I).

### Subgroup and sensitive analysis

The stratified analyses revealed TyG-related indices had stronger associations with all-cause mortality of the MetS population with the clinical characteristics of non-Hispanic Black race, normal or overweight BMI, and married or living with a partner (Figure S1-S3). Additionally, subgroup analyses revealed some significant interaction effects (*p* < 0.05), indicating that the relationship between TyG-related indices and all-cause mortality of the MetS population varied significantly across different subgroups. Specifically, the effect of TyG-related indices on all-cause mortality of the MetS population differed among subgroups defined by age, gender, race, marital status, history of cancer, and serum levels of ALB, ALT, AST, BUN, TC, and UA. (Figure S1-S3). To check the robustness of the main findings, several sensitive analyses were performed. First, after excluding the participants with a diagnosis of diabetes mellitus at baseline, the TyG-related indices remained significantly associated with diabetes mortality during the follow-up (**Table S7-S9**). After excluding the participants who died within 2 years after the survey, we determined consistent associations between TyG-related indices with the all-cause as well as cause-specific mortality risk among the modified MetS population (**Table S10-S12**). Similarly, the results were still robust in the study population without a history of comorbidities (**Table S13-S15**) or with a high BMI index (**Table S16-S18**).

## Discussion

In the current study, we preliminary observed the association between a series of TyG-related indices with the all-cause and cause-specific mortality of the MetS population, based on one large-scale, population-based cohort. High levels of TyG and TyG-WHtR indices are independently associated with an increased risk of all-cause mortality in the MetS population. Meanwhile, the TyG-WC and TyG-WHtR indices maintained pronounced roles in predicting the cardiovascular mortality of the MetS population. Alternatively, all of the TyG-related indices presented strong relationships with the diabetes mortality of the MetS population. The RCS curves displayed different linear as well as non-linear associations between TyG-related indices with all-cause and cause-specific mortality of the MetS population. Our findings indicated that TyG-related indices might be one of the useful surrogate biomarkers for the clinical follow-up management of the MetS population.

MetS accounts for a non-negligible proportion of the general population, which would significantly increase the risk for multiple diseases and impair health outcomes in later life. Therefore, identifying the potential risk and prognostic factors would bring cost-saving effects on the management of this particular population [38]. Current evidence suggests that chronic insulin resistance plays a key role in the onset of MetS. For this reason, pathophysiological factors related to insulin resistance were observed in the metabolic disorders of the MetS population. The hyperinsulinemia-euglycemic clamp is the most accurate method for the diagnosis of insulin resistance. Nevertheless, it is rarely used in clinical care owing to the obstacles of medical cost, accessibility, and reproducibility in resource-limited countries or regions. Alternatively, homeostatic model assessment of insulin resistance (HOMA-IR) and TyG index were recently identified and proved to be reliable tools for insulin-resistant screening. Regarding the applications of these surrogate biomarkers in screening the MetS, noteworthy progress has been made. Notably, the large-scale, population-based evidence determined that the TyG index showed higher predictive power for predicting the prevalence of MetS in the general population from different countries or races [14, 16, 18, 39]. Furthermore, the latest systemic review and meta-analysis of 49,325 participants derived from thirteen observational studies supported the clinical utility of the TyG index in the screening of MetS, with optimal sensitivity and specificity [19].

Although different studies have highlighted the promising predictive role of the TyG index in the onset of MetS, only a few studies have explored the prognosis role of the TyG index on the MetS population. If the robust link between the TyG index with the prognosis of the MetS population Therefore, our study filled this important research gap and took it further. To our knowledge, we were the first study that determined the independent prognostic role of the TyG index among the U.S. population with MetS. Participants with high quartile levels of the TyG index showed an increased risk for all-cause mortality and diabetes mortality. Recently, a positive association between the TyG index with the survival of patients in different disease settings was observed [40–43]. For instance, Zhou and colleagues conducted a retrospective study focusing on the association between the TyG index with the mortality risk of patients with chronic heart failure. In their analysis, high levels of TyG index were associated with nearly 2-fold increased risks for all-cause and cardiovascular mortality of the patients [23]. More importantly, the predictive ability of the TyG index was observed to be more pronounced among patients with MetS at the subgroup analysis. Interestingly, our results found that the TyG index showed a weak correlation with cardiovascular mortality among the MetS population (adjusted *p* = 0.060). By contrast, our study determined that the TyG-WHtR index showed a significant correlation with cardiovascular mortality among the MetS population. Previously, BMI was a simple, easily available anthropometric measurement for accounting height-adjusted weight of individuals [44]. However, recent evidence derived from different studies recognized the paradox of BMI in reflecting the obesity and mortality risk of the population [44, 45]. Notably, the large-scale epidemiological study demonstrated that a considerable proportion of the U.S. population (nearly 30% of males and 50% of females) had abdominal obesity, even though their BMI was in the healthy range [46]. By contrast, the enlarged WC frequently predicted increased fat mass accumulation, which could adequately reflect the real burden of central obesity [47, 48]. Accordingly, WC showed superior predicting value for predicting insulin resistance and diagnosis of adult MetS when compared with BMI [2, 8, 49, 50]. Besides, the WC was not only significantly associated with the development of complications under the MetS [51, 52] but also linked to the mortality risk in the general population [48, 53–56]. Furthermore, compared with other obesity-related indicators, the WHtR index was validated to present significantly better prediction power for cardiometabolic health and mortality, especially among the diabetes population [57–59]. Especially, Dang et al. observed the enhanced predictive power of the TyG-WHtR index in cardiovascular diseases and related mortality risk among the general population when compared with the TyG index [25]. Therefore, our results combined with previous findings highlight that the choice of TyG-related indices for predicting the mortality of patients should be tailored to the particular disease condition.

While emerging clinical epidemiological studies have proved the good predicting performance of the TyG index in the onset and progress of diseases, the exact underlying biological mechanisms remained less studied. Compelling evidence indicated that insulin resistance was closely related to endothelial dysfunction, oxidative stress, and inflammatory response of the systemic metabolism [60–62]. As early signs of insulin resistance, TyG-related indices could help to inflect the pro-inflammatory condition of the participants and indicate the vulnerability to the severity and progress of diseases. Of note, one non-negligible question that was raised in recent studies was determining the best cut-off point for TyG-related indices among different stratified populations [11, 13]. Especially, Zhang et al. identified that the cutoff point in predicting the mortality risk for cardiovascular participants with diabetes or pre-diabetes was 9.05. In our study, the RCS curve revealed that the threshold value of the TyG index for predicting the all-cause mortality of the MetS population was around 7.47, which indicated the variation of the TyG index in predicting the survival of participants with varied disease conditions. Thus, further prospective longitudinal studies are warranted to develop and comprehensively evaluate the adequate cut-off point of new insulin resistance biomarkers for various ethnicities associated with the risk of developing comorbidities in later life and provide additional evidence in the updating of available clinical guidelines.

### Strengths and limitations

Some noteworthy strengths of the present study should be highlighted. Initially, this is the first study focusing on comprehensively evaluation the associations between TyG-related indices with the all-cause as well as cause-specific mortality of the MetS population. Our results highlight the promising application prospect of TyG-related indices in the clinical management of MetS, the group suffering from systemic metabolic disorders. Second, the prospective, population-based study design helps us to determine the robust evidence between the TyG index and mortality of the MetS population. Last, the sensitive analysis shows consistent associations as we determine in the main findings.

Nevertheless, there are some limitations existing in the present study. First, while the present study was conducted based on a large-scale, population-based cohort, the data of blood indicators and anthropometric measurements were only collected at the baseline. Whether the trajectories of TyG-related indices would offer valuable insights into the clinical management of individuals with MetS is worth exploring. Second, the diagnosis of MetS was solely based on the NCEP III criteria, which might allow us to underestimate its incidence among different populations. Additionally, while we controlled a series of covariates including socioeconomic status, comorbidities, blood tests, and personal habits, some residual confounders (such as inherent genetic heterogeneity and changes of lifestyle during the lifetime) were observed to play a role in the MetS were unavailable in the present database. Besides, the smoking status was based on a categorical variable instead of pack-per year might also result in residual bias. Of note, the findings we determined need to be interpreted cautiously due to the identification of negative confounders. The observational study design and imbalanced baseline clinical characteristics might contribute to these confounding factors during the analyses. Future well-designed, longitudinal, propensity score-matched works are warranted to validate our findings and mitigate the impact of negative confounders. Last, although we observed the association between the TyG-related indices and mortality risk among the U.S. MetS population, its practical clinical application in different phenotypes of MetS population and varied races from other countries needs to be further validated.

## Conclusion

Summarily, TyG-related indices showed significant correlations with all-cause and cause-specific mortality of the MetS population. The TyG index showed a strong association with diabetes mortality, whereas the TyG-WC and TyG-WHtR indices were pronounced associated with cardiovascular and diabetes mortality risks among the MetS population. Our findings demonstrated the application prospect of TyG-related indices in the management of the MetS population during the long-term follow-up, which would help endocrinologists to make better clinical care for the MetS population. Future longitudinal works are warranted to validate our findings.

### Electronic supplementary material

Below is the link to the electronic supplementary material.

Supplementary Material 1

## Data Availability

The datasets generated during and/or analyzed during the current study are available from the corresponding author upon reasonable request.
