# Supplementary material for: Association between triglyceride-glucose related indices with the all-cause and cause-specific mortality among the population with metabolic syndrome
Source: Cardiovasc Diabetol. 2024 Apr 24;23:134. doi: 10.1186/s12933-024-02215-0 (PMC11044377; doi:10.1186/s12933-024-02215-0)
Supplement: Supplementary file 1 — Supplementary Material 1 [file 12933_2024_2215_MOESM1_ESM.docx]

**Supplementary files**

**Association between Triglyceride-Glucose related Indices with the All-cause and Cause-specific Mortality among the Population with Metabolic Syndrome**

Xiaoyuan Wei ^1#^, M.D., Yu Min ^2#^, M.D., Ge Song ^2#^, M.D., Xin Ye ^3^, M.D., Lei Liu^1*^, Ph.D.

^1^ ^1^ Department of Head and Neck Oncology, West China Hospital, Sichuan University, Chengdu, 610041, P.R. China.

^2^ Department of Biotherapy, West China Hospital, Sichuan University, Chengdu 610041, P.R. China.

^2^ Department of Oncology, Chengdu University of Traditional Chinese Medicine, Chengdu 610041, P.R. China.

*Corresponding Authors:

Prof. Lei Liu is to be contacted at the Department of Head and Neck Oncology, Cancer Center and State Key Laboratory of Biotherapy, West China Hospital, Sichuan University, Chengdu 610041, Sichuan, China. E-mail address: [liuleihx@gmail.com](mailto:liuleihx@gmail.com).

^#^ Xiaoyuan Wei, Yu Min, and Ge Song contributed equally to this work.

**Figure S1.** The subgroup analysis between the TyG index with all-cause mortality of MetS population. TyG: triglyceride-glucose; Q: quartile; HR: hazard ratio; CI: confidence interval; MetS: metabolic syndrome; TC: total cholesterol; CKD: chronic kidney disease; CVD: cardiovascular disease; eGFR: estimated glomerular filtration rate; BUN: blood urea nitrogen; HDL-C: high-density lipoprotein cholesterol; LDL-C: low-density lipoprotein cholesterol; UA: uric acid; ALT: glutamic-pyruvic transaminase; AST: aspartate transaminase; ALB: albumin; BMI: body mass index; TBil: total bilirubin.


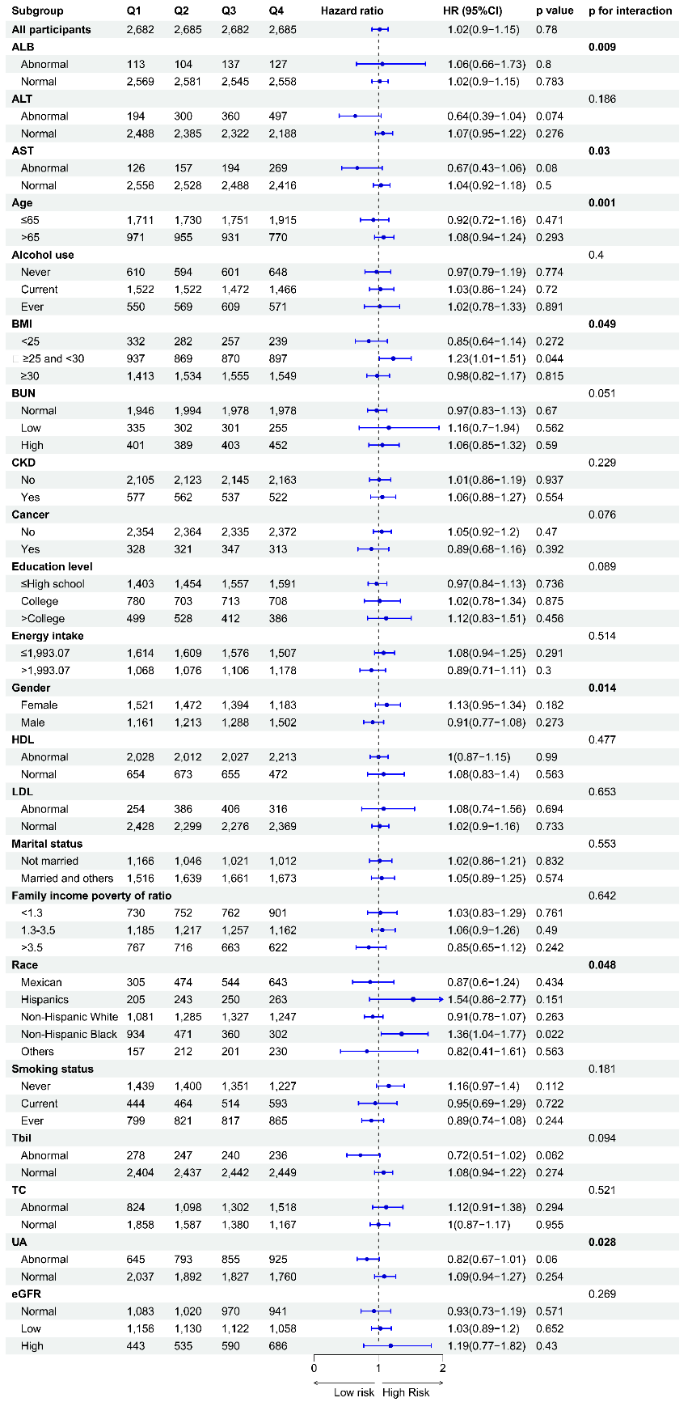


**Figure S2.** The subgroup analysis between the TyG-WC index with all-cause mortality of MetS population. TyG-WC: TyG combining with waist circumference; Q: quartile; HR: hazard ratio; CI: confidence interval; MetS: metabolic syndrome; TC: total cholesterol; CKD: chronic kidney disease; CVD: cardiovascular disease; eGFR: estimated glomerular filtration rate; BUN: blood urea nitrogen; HDL-C: high-density lipoprotein cholesterol; LDL-C: low-density lipoprotein cholesterol; UA: uric acid; ALT: glutamic-pyruvic transaminase; AST: aspartate transaminase; ALB: albumin; BMI: body mass index; TBil: total bilirubin.


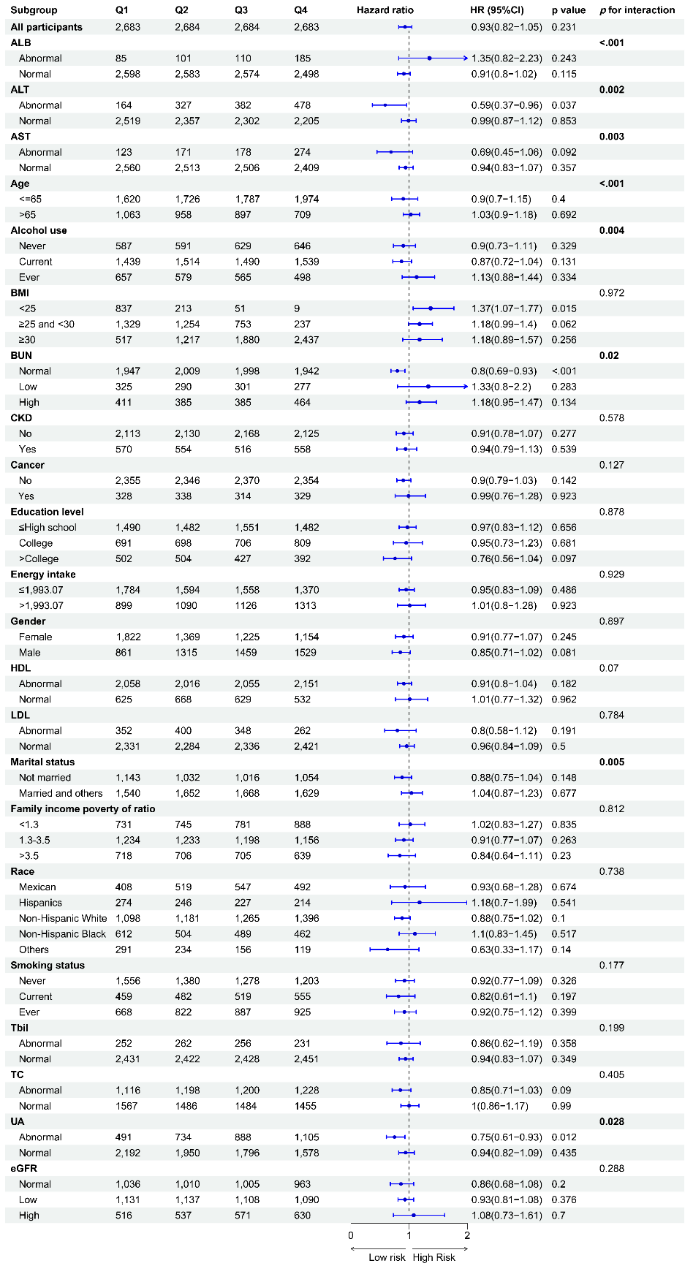


**Figure S3.** The subgroup analysis between the TyG-WHtR index with all-cause mortality of the MetS population. TyG-WC: TyG combining with waist to height ratio; Q: quartile; HR: hazard ratio; CI: confidence interval; MetS: metabolic syndrome; TC: total cholesterol; CKD: chronic kidney disease; CVD: cardiovascular disease; eGFR: estimated glomerular filtration rate; BUN: blood urea nitrogen; HDL-C: high-density lipoprotein cholesterol; LDL-C: low-density lipoprotein cholesterol; UA: uric acid; ALT: glutamic-pyruvic transaminase; AST: aspartate transaminase; ALB: albumin; BMI: body mass index; TBil: total bilirubin.


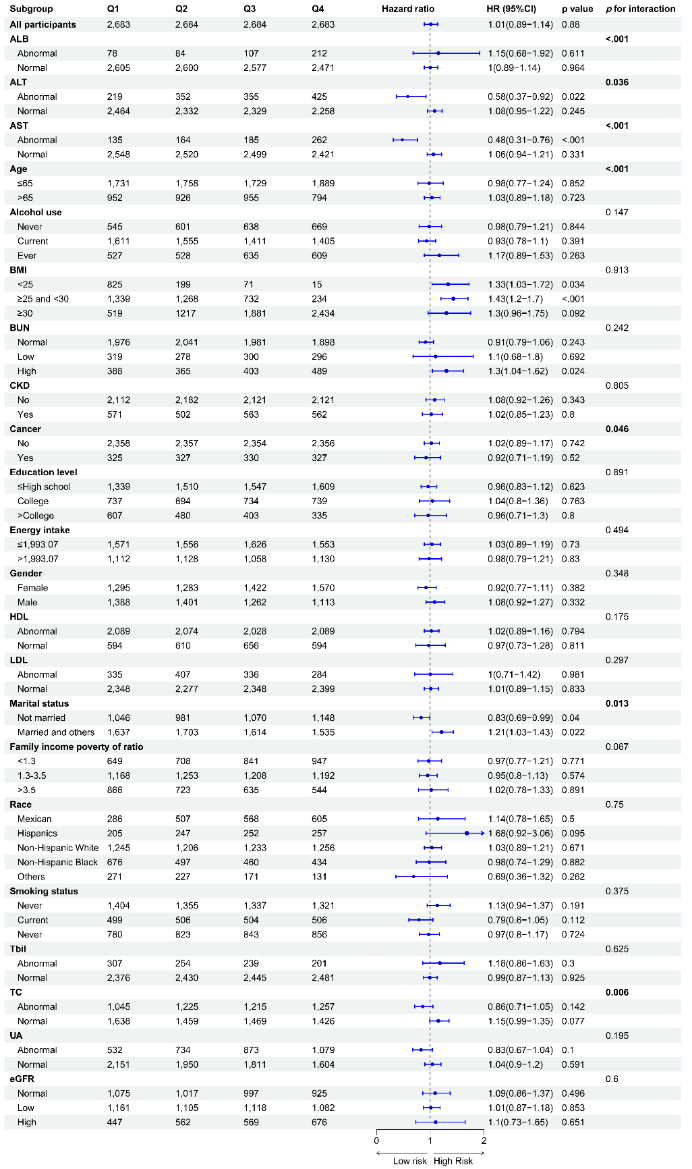


**Table S1.** The demographic characteristics of the MetS population were stratified by varied quartiles of the TyG index.

| **Variable** | **Total (n = 10,734)** | **Quartiles of TyG index** | | | | **P^*^** |
| --- | --- | --- | --- | --- | --- | --- |
|  |  | **Q1(5.14 – 7.01)**  **(n = 2,682)** | **Q2(7.01 – 7.41)**  **(n = 2,685)** | **Q3(7.41 – 7.80)**  **(n = 2,682)** | **Q4(7.80 – 11.76)**  **(n = 2,685)** |  |
| Age, M (Q₁, Q₃) | 59.00 (45.00, 70.00) | 60.00 (46.00,70.00) | 60.00 (45.00,70.00) | 59.50 (44.00,70.00) | 57.00 (44.00,67.00) | **<.001** |
| Energy intake, M (Q₁, Q₃) | 1848.00 (1384.25, 2423.00) | 1848.00 (1369.00,2369.00) | 1848.00 (1372.00,2406.00) | 1848.00 (1376.25,2421.75) | 1850.00 (1437.00,2504.00) | **<.001** |
| FBG, M (Q₁, Q₃) | 108.00 (100.50, 124.00) | 104.00 (98.00,111.00) | 106.00 (100.00,116.00) | 108.90 (101.00,123.72) | 126.00 (106.00,184.00) | **<.001** |
| TC, M (Q₁, Q₃) | 5.04 (4.32, 5.84) | 4.68 (3.98,5.41) | 4.94 (4.27,5.71) | 5.17 (4.45,5.90) | 5.43 (4.66,6.26) | **<.001** |
| TG, M (Q₁, Q₃) | 1.65 (1.12, 2.29) | 0.88 (0.72,1.04) | 1.42 (1.25,1.63) | 1.99 (1.74,2.23) | 2.98 (2.40,3.86) | **<.001** |
| HDL-C, M (Q₁, Q₃) | 45.00 (39.00, 54.00) | 52.00 (44.00,66.00) | 46.00 (40.00,56.00) | 45.00 (39.00,51.00) | 41.00 (34.00,46.00) | **<.001** |
| LDL-C, M (Q₁, Q₃) | 2.97 (2.38, 3.58) | 2.85 (2.22,3.44) | 3.00 (2.43,3.67) | 3.05 (2.46,3.70) | 2.97 (2.46,3.46) | **<.001** |
| BUN, M (Q₁, Q₃) | 4.64 (3.93, 6.07) | 4.64 (3.57,6.07) | 4.64 (3.93,6.07) | 4.64 (3.93,6.07) | 5.00 (3.93,6.10) | **0.003** |
| UA, M (Q₁, Q₃) | 345.00 (285.50, 404.50) | 327.10 (273.60,380.70) | 339.00 (291.50,398.50) | 350.90 (291.50,404.50) | 356.90 (291.50,416.40) | **<.001** |
| eGFR, M (Q₁, Q₃) | 87.10 (64.89, 113.80) | 85.08 (63.97,107.66) | 86.46 (64.00,112.16) | 87.17 (65.39,115.82) | 90.92 (66.24,120.81) | **<.001** |
| ALT, M (Q₁, Q₃) | 22.00 (17.00, 30.75) | 20.00 (16.00,26.00) | 21.00 (17.00,29.00) | 22.00 (17.00,31.00) | 25.00 (19.00,36.00) | **<.001** |
| AST, M (Q₁, Q₃) | 23.00 (19.00, 28.00) | 22.00 (19.00,27.00) | 23.00 (19.00,27.00) | 23.00 (19.00,28.00) | 24.00 (19.00,30.00) | **<.001** |
| ALB, M (Q₁, Q₃) | 42.00 (39.00, 44.00) | 41.00 (39.00,43.00) | 42.00 (39.00,44.00) | 42.00 (40.00,44.00) | 42.00 (40.00,44.00) | **<.001** |
| TBil, M (Q₁, Q₃) | 10.26 (8.55, 13.68) | 10.30 (8.55,13.68) | 10.26 (8.55,13.68) | 10.26 (8.55,13.68) | 10.26 (8.55,13.68) | 0.039 |
| Gender, n (%) |  |  |  |  |  | **<.001** |
| Female | 5,570 (51.89) | 1,521 (56.71) | 1,472 (54.82) | 1,394 (51.98) | 1,183 (44.06) |  |
| Male | 5,164 (48.11) | 1,161 (43.29) | 1,213 (45.18) | 1,288 (48.02) | 1,502 (55.94) |  |
| Race, n (%) |  |  |  |  |  | **<.001** |
| Mexican | 1,966 (18.32) | 305 (11.37) | 474 (17.65) | 544 (20.28) | 643 (23.95) |  |
| Hispanics | 961 (8.95) | 205 (7.64) | 243 (9.05) | 250 (9.32) | 263 (9.80) |  |
| Non-Hispanic White | 4,940 (46.02) | 1081 (40.31) | 1285 (47.86) | 1327 (49.48) | 1247 (46.44) |  |
| Non-Hispanic Black | 2,067 (19.26) | 934 (34.82) | 471 (17.54) | 360 (13.42) | 302 (11.25) |  |
| Others | 800 (7.45) | 157 (5.85) | 212 (7.90) | 201 (7.49) | 230 (8.57) |  |
| Education level, n (%) |  |  |  |  |  | **<.001** |
| ≤High school | 6,005 (55.94) | 1,403 (52.31) | 1,454 (54.15) | 1,557 (58.05) | 1,591 (59.26) |  |
| College | 2,904 (27.05) | 780 (29.08) | 703 (26.18) | 713 (26.58) | 708 (26.37) |  |
| >College | 1,825 (17) | 499 (18.61) | 528 (19.66) | 412 (15.36) | 386 (14.38) |  |
| Marital status, n (%) |  |  |  |  |  | **<.001** |
| Not married | 4,245 (39.55) | 1,166 (43.48) | 1,046 (38.96) | 1,021 (38.07) | 1,012 (37.69) |  |
| Married or living with a partner | 6,489 (60.45) | 1,516 (56.52) | 1,639 (61.04) | 1,661 (61.93) | 1,673 (62.31) |  |
| Poverty income ratio, n (%) |  |  |  |  |  | **<.001** |
| <1.3 | 3,145 (29.3) | 730 (27.22) | 752 (28.01) | 762 (28.41) | 901 (33.56) |  |
| 1.3-3.5 | 4,821 (44.91) | 1,185 (44.18) | 1,217 (45.33) | 1,257 (46.87) | 1,162 (43.28) |  |
| >3.5 | 2,768 (25.79) | 767 (28.60) | 716 (26.67) | 663 (24.72) | 622 (23.17) |  |
| BMI, n (%) |  |  |  |  |  | **<.001** |
| <25 | 1,110 (10.34) | 332 (12.38) | 282 (10.50) | 257 (9.58) | 239 (8.90) |  |
| ≥25 and <30 | 3,573 (33.29) | 937 (34.94) | 869 (32.36) | 870 (32.44) | 897 (33.41) |  |
| ≥30 | 6,051 (56.37) | 1,413 (52.68) | 1,534 (57.13) | 1,555 (57.98) | 1,549 (57.69) |  |
| Smoking status, n (%) |  |  |  |  |  | **<.001** |
| Never | 5,417 (50.47) | 1,439 (53.65) | 1,400 (52.14) | 1,351 (50.37) | 1,227 (45.70) |  |
| Current | 2,015 (18.77) | 444 (16.55) | 464 (17.28) | 514 (19.16) | 593 (22.09) |  |
| Ever | 3,302 (30.76) | 799 (29.79) | 821 (30.58) | 817 (30.46) | 865 (32.22) |  |
| Alcohol use, n (%) |  |  |  |  |  | 0.245 |
| Never | 2,453 (22.85) | 610 (22.74) | 594 (22.12) | 601 (22.41) | 648 (24.13) |  |
| Current | 5,982 (55.73) | 1,522 (56.75) | 1,522 (56.69) | 1,472 (54.88) | 1,466 (54.60) |  |
| Ever | 2,299 (21.42) | 550 (20.51) | 569 (21.19) | 609 (22.71) | 571 (21.27) |  |
| Cancer, n (%) |  |  |  |  |  | 0.521 |
| No | 9,425 (87.81) | 2,354 (87.77) | 2,364 (88.04) | 2,335 (87.06) | 2,372 (88.34) |  |
| Yes | 1,309 (12.19) | 328 (12.23) | 321 (11.96) | 347 (12.94) | 313 (11.66) |  |
| CKD, n (%) |  |  |  |  |  | 0.239 |
| No | 8,536 (79.52) | 2,105 (78.49) | 2,123 (79.07) | 2,145 (79.98) | 2,163 (80.56) |  |
| Yes | 2,198 (20.48) | 577 (21.51) | 562 (20.93) | 537 (20.02) | 522 (19.44) |  |
| CVD, n (%) |  |  |  |  |  | 0.064 |
| No | 9,180 (85.52) | 2,299 (85.72) | 2,305 (85.85) | 2,320 (86.50) | 2,256 (84.02) |  |
| Yes | 1,554 (14.48) | 383 (14.28) | 380 (14.15) | 362 (13.50) | 429 (15.98) |  |
| All-cause Mortality, n (%) |  |  |  |  |  | **0.002** |
| No | 8,519 (79.36) | 2,176 (81.13) | 2,140 (79.70) | 2,136 (79.64) | 2,067 (76.98) |  |
| Yes | 2,215 (20.64) | 506 (18.87) | 545 (20.30) | 546 (20.36) | 618 (23.02) |  |
| Diabetes Mortality, n (%) |  |  |  |  |  | **<.001** |
| No | 10,374 (96.65) | 2,635 (98.25) | 2,614 (97.36) | 2,607 (97.20) | 2,518 (93.78) |  |
| Yes | 360 (3.35) | 47 (1.75) | 71 (2.64) | 75 (2.80) | 167 (6.22) |  |
| Cardiovascular Mortality, n (%) |  |  |  |  |  | 0.407 |
| No | 10,119 (94.27) | 2,539 (94.67) | 2,540 (94.60) | 2,523 (94.07) | 2,517 (93.74) |  |
| Yes | 615 (5.73) | 143 (5.33) | 145 (5.40) | 159 (5.93) | 168 (6.26) |  |

^*^P-value <0.017 was considered significant, as we had to correct our analysis for multiple testing (P-value of 0.017 was calculated as: 0.05 divided by 3).

Abbreviation: MetS: metabolic syndrome; M: median; Q: quartile; Q_1:_ 1^st^ quartile; Q_3:_ 3^rd^ quartile; n: number; FBG: fasting blood glucose; TyG: triglyceride-glucose; TC: total cholesterol; TG: triglyceride; CKD: chronic kidney disease; CVD: cardiovascular disease; eGFR: estimated glomerular filtration rate, BUN: blood urea nitrogen; HDL-C: high-density lipoprotein cholesterol; LDL-C: low-density lipoprotein cholesterol; UA: uric acid; ALT: glutamic-pyruvic transaminase; AST: aspartate transaminase; ALB: albumin; TBil: total bilirubin; BMI: body mass index.

**Table S2**. The demographic characteristics of the MetS population were stratified by varied quartiles of the TyG-WC index.

| **Variable** | **Total (n = 10,734)** | **Quartiles of TyG-WC index** | | | | **P^*^** |
| --- | --- | --- | --- | --- | --- | --- |
|  |  | **Q1(440.81 – 711.74)**  **(n = 2,683)** | **Q2 (711.74 – 787.28)**  **(n = 2,684)** | **Q3(787.28 – 876.29)**  **(n = 2,684)** | **Q4 (876.29 –1489.27)**  **(n = 2,683)** |  |
| Age, M (Q₁, Q₃) | 59.00 (45.00, 70.00) | 61.00 (47.00,72.00) | 60.00 (46.00,70.00) | 59.00 (44.00,69.25) | 56.00 (43.00,66.00) | **<.001** |
| Energy intake, M (Q₁, Q₃) | 1848.00 (1384.25, 2423.00) | 1747.00 (1319.00,2201.50) | 1848.00 (1371.32,2388.25) | 1848.00 (1410.75,2485.53) | 1964.00 (1478.50,2629.50) | **<.001** |
| FBG, M (Q₁, Q₃) | 108.00 (100.50, 124.00) | 104.00 (98.00,111.40) | 106.00 (100.00,117.00) | 109.35 (101.00,126.40) | 119.00 (105.00,162.00) | **<.001** |
| TC, M (Q₁, Q₃) | 5.04 (4.32, 5.84) | 4.96 (4.24,5.77) | 5.07 (4.32,5.83) | 5.04 (4.29,5.87) | 5.09 (4.40,5.90) | **<.001** |
| TG, M (Q₁, Q₃) | 1.65 (1.12, 2.29) | 1.08 (0.80,1.57) | 1.50 (1.11,2.00) | 1.85 (1.35,2.47) | 2.20 (1.60,3.22) | **<.001** |
| HDL-C, M (Q₁, Q₃) | 45.00 (39.00, 54.00) | 51.00 (44.00,65.00) | 45.00 (40.00,55.00) | 45.00 (38.00,50.00) | 42.00 (35.00,48.00) | **<.001** |
| LDL-C, M (Q₁, Q₃) | 2.97 (2.38, 3.58) | 2.97 (2.35,3.62) | 2.97 (2.43,3.67) | 2.97 (2.38,3.60) | 2.97 (2.38,3.41) | **<.001** |
| BUN, M (Q₁, Q₃) | 4.64 (3.93, 6.07) | 4.64 (3.60,6.07) | 4.64 (3.90,6.07) | 4.64 (3.93,6.07) | 5.00 (3.93,6.40) | **0.008** |
| UA, M (Q₁, Q₃) | 345.00 (285.50, 404.50) | 309.30 (261.70,356.90) | 339.00 (285.50,398.50) | 356.90 (303.30,410.40) | 368.80 (315.20,434.20) | **<.001** |
| eGFR, M (Q₁, Q₃) | 87.10 (64.89, 113.80) | 86.42 (64.34,110.93) | 85.77 (64.47,111.83) | 87.46 (65.80,114.55) | 88.48 (64.50,117.50) | **0.009** |
| ALT, M (Q₁, Q₃) | 22.00 (17.00, 30.75) | 19.00 (15.00,25.00) | 22.00 (17.00,30.00) | 23.00 (18.00,33.00) | 25.00 (18.00,35.00) | **<.001** |
| AST, M (Q₁, Q₃) | 23.00 (19.00, 28.00) | 22.00 (19.00,27.00) | 23.00 (19.00,28.00) | 23.00 (19.00,28.00) | 23.00 (19.00,29.00) | **<.001** |
| ALB, M (Q₁, Q₃) | 42.00 (39.00, 44.00) | 42.00 (40.00,44.00) | 42.00 (40.00,44.00) | 42.00 (40.00,44.00) | 41.00 (39.00,43.00) | **<.001** |
| TBil, M (Q₁, Q₃) | 10.26 (8.55, 13.68) | 10.26 (8.55,13.68) | 10.30 (8.55,13.68) | 10.26 (8.55,13.68) | 10.26 (8.55,13.68) | **<.001** |
| Gender, n (%) |  |  |  |  |  | **<.001** |
| Female | 5,570 (51.89) | 1,822 (67.91) | 1,369 (51.01) | 1,225 (45.64) | 1,154 (43.01) |  |
| Male | 5,164 (48.11) | 861 (32.09) | 1,315 (48.99) | 1,459 (54.36) | 1,529 (56.99) |  |
| Race, n (%) |  |  |  |  |  | **<.001** |
| Mexican | 1,966 (18.32) | 408 (15.21) | 519 (19.34) | 547 (20.38) | 492 (18.34) |  |
| Hispanics | 961 (8.95) | 274 (10.21) | 246 (9.17) | 227 (8.46) | 214 (7.98) |  |
| Non-Hispanic White | 4,940 (46.02) | 1098 (40.92) | 1181 (44.00) | 1265 (47.13) | 1396 (52.03) |  |
| Non-Hispanic Black | 2,067 (19.26) | 612 (22.81) | 504 (18.78) | 489 (18.22) | 462 (17.22) |  |
| Others | 800 (7.45) | 291 (10.85) | 234 (8.72) | 156 (5.81) | 119 (4.44) |  |
| Education level, n (%) |  |  |  |  |  | **<.001** |
| ≤High school | 6,005 (55.94) | 1,490 (55.53) | 1,482 (55.22) | 1,551 (57.79) | 1,482 (55.24) |  |
| College | 2,904 (27.05) | 691 (25.75) | 698 (26.01) | 706 (26.30) | 809 (30.15) |  |
| >College | 1,825 (17) | 502 (18.71) | 504 (18.78) | 427 (15.91) | 392 (14.61) |  |
| Marital status, n (%) |  |  |  |  |  | **0.002** |
| Not married | 4,245 (39.55) | 1,143 (42.60) | 1,032 (38.45) | 1,016 (37.85) | 1,054 (39.28) |  |
| Married or living with a partner | 6,489 (60.45) | 1,540 (57.40) | 1,652 (61.55) | 1,668 (62.15) | 1,629 (60.72) |  |
| Poverty income ratio, n (%) |  |  |  |  |  | **<.001** |
| <1.3 | 3,145 (29.3) | 731 (27.25) | 745 (27.76) | 781 (29.10) | 888 (33.10) |  |
| 1.3-3.5 | 4,821 (44.91) | 1,234 (45.99) | 1,233 (45.94) | 1,198 (44.63) | 1,156 (43.09) |  |
| >3.5 | 2,768 (25.79) | 718 (26.76) | 706 (26.30) | 705 (26.27) | 639 (23.82) |  |
| BMI, n (%) |  |  |  |  |  | **<.001** |
| <25 | 1110 (10.34) | 837 (31.20) | 213 (7.94) | 51 (1.90) | 9 (0.34) |  |
| ≥25 and <30 | 3,573 (33.29) | 1,329 (49.53) | 1,254 (46.72) | 753 (28.06) | 237 (8.83) |  |
| ≥30 | 6,051 (56.37) | 517 (19.27) | 1,217 (45.34) | 1,880 (70.04) | 2,437 (90.83) |  |
| Smoking status, n (%) |  |  |  |  |  | **<.001** |
| Never | 5,417 (50.47) | 1,556 (57.99) | 1,380 (51.42) | 1,278 (47.62) | 1,203 (44.84) |  |
| Current | 2,015 (18.77) | 459 (17.11) | 482 (17.96) | 519 (19.34) | 555 (20.69) |  |
| Ever | 3,302 (30.76) | 668 (24.90) | 822 (30.63) | 887 (33.05) | 925 (34.48) |  |
| Alcohol use, n (%) |  |  |  |  |  | **<.001** |
| Never | 2,453 (22.85) | 587 (21.88) | 591 (22.02) | 629 (23.44) | 646 (24.08) |  |
| Current | 5,982 (55.73) | 1,439 (53.63) | 1,514 (56.41) | 1,490 (55.51) | 1,539 (57.36) |  |
| Ever | 2,299 (21.42) | 657 (24.49) | 579 (21.57) | 565 (21.05) | 498 (18.56) |  |
| Cancer, n (%) |  |  |  |  |  | 0.795 |
| No | 9,425 (87.81) | 2,355 (87.77) | 2,346 (87.41) | 2,370 (88.30) | 2,354 (87.74) |  |
| Yes | 1,309 (12.19) | 328 (12.23) | 338 (12.59) | 314 (11.70) | 329 (12.26) |  |
| CKD, n (%) |  |  |  |  |  | 0.288 |
| No | 8,536 (79.52) | 2,113 (78.76) | 2,130 (79.36) | 2,168 (80.77) | 2,125 (79.20) |  |
| Yes | 2,198 (20.48) | 570 (21.24) | 554 (20.64) | 516 (19.23) | 558 (20.80) |  |
| CVD, n (%) |  |  |  |  |  | **<.001** |
| No | 9,180 (85.52) | 2,344 (87.36) | 2,316 (86.29) | 2,296 (85.54) | 2,224 (82.89) |  |
| Yes | 1,554 (14.48) | 339 (12.64) | 368 (13.71) | 388 (14.46) | 459 (17.11) |  |
| All-cause mortality, n (%) |  |  |  |  |  | 0.468 |
| No | 8,519 (79.36) | 2,131 (79.43) | 2,148 (80.03) | 2,103 (78.35) | 2,137 (79.65) |  |
| Yes | 2,215 (20.64) | 552 (20.57) | 536 (19.97) | 581 (21.65) | 546 (20.35) |  |
| Diabetes Mortality, n (%) |  |  |  |  |  | **<.001** |
| No | 10,374 (96.65) | 2,626 (97.88) | 2,617 (97.50) | 2,586 (96.35) | 2,545 (94.86) |  |
| Yes | 360 (3.35) | 57 (2.12) | 67 (2.50) | 98 (3.65) | 138 (5.14) |  |
| Cardiovascular Mortality, n (%) |  |  |  |  |  | 0.303 |
| No | 10,119 (94.27) | 2,538 (94.60) | 2,541 (94.67) | 2,529 (94.23) | 2,511 (93.59) |  |
| Yes | 615 (5.73) | 145 (5.40) | 143 (5.33) | 155 (5.77) | 172 (6.41) |  |

^*^P-value <0.017 was considered significant, as we had to correct our analysis for multiple testing (P-value of 0.017 was calculated as: 0.05 divided by 3).

Abbreviation: MetS: metabolic syndrome; M: median; Q: quartile; Q_1:_ 1^st^ quartile; Q_3:_ 3^rd^ quartile; n: number; FBG: fasting blood glucose; TyG-WC: TyG combining with waist circumference; TC: total cholesterol; TG: triglyceride; CKD: chronic kidney disease; CVD: cardiovascular disease; eGFR: estimated glomerular filtration rate; BUN: blood urea nitrogen; HDL-C: high-density lipoprotein cholesterol; LDL-C: low-density lipoprotein cholesterol; UA: uric acid; ALT: glutamic-pyruvic transaminase; AST: aspartate transaminase; ALB: albumin; Tbil: total bilirubin; BMI: body mass index.

**Table S3**. The demographic characteristics of the MetS population were stratified by varied quartiles of the TyG-WHtR index.

| **Variable** | **Total (n = 10,734)** | **Quartiles of TyG-WHtR index** | | | | **P^*^** |
| --- | --- | --- | --- | --- | --- | --- |
|  |  | **Q1(2.38 – 4.28)**  **(n = 2,683)** | **Q2 (4.28 – 4.72)**  **(n = 2,684)** | **Q3(4.72 – 5.25)**  **(n = 2,684)** | **Q4 (5.25 – 8.91)**  **(n = 2,683)** |  |
| Age, M (Q₁, Q₃) | 59.00 (45.00, 70.00) | 60.00 (46.00,71.00) | 59.00 (44.00,70.00) | 60.00 (46.00,70.00) | 57.00 (44.00,68.00) | **<.001** |
| Energy intake, M (Q₁, Q₃) | 1848.00 (1384.25, 2423.00) | 1848.00 (1423.00,2384.50) | 1848.00 (1388.89,2440.50) | 1848.00 (1357.00,2414.50) | 1848.00 (1372.00,2446.00) | 0.325 |
| FBG, M (Q₁, Q₃) | 108.00 (100.50, 124.00) | 104.00 (99.00,112.00) | 106.00 (100.00,116.00) | 109.50 (101.00,126.00) | 120.00 (105.00,163.50) | **<.001** |
| TC, M (Q₁, Q₃) | 5.04 (4.32, 5.84) | 4.89 (4.16,5.69) | 5.07 (4.34,5.84) | 5.07 (4.37,5.90) | 5.12 (4.40,5.92) | **<.001** |
| TG, M (Q₁, Q₃) | 1.65 (1.12, 2.29) | 1.07 (0.80,1.48) | 1.57 (1.13,2.08) | 1.85 (1.35,2.47) | 2.16 (1.58,3.14) | **<.001** |
| HDL-C, M (Q₁, Q₃) | 45.00 (39.00, 54.00) | 49.00 (42.00,63.50) | 45.00 (39.00,55.00) | 45.00 (39.00,52.00) | 43.00 (36.00,49.00) | **<.001** |
| LDL-C, M (Q₁, Q₃) | 2.97 (2.38, 3.58) | 2.97 (2.33,3.60) | 3.00 (2.43,3.70) | 2.97 (2.40,3.57) | 2.97 (2.38,3.46) | **<.001** |
| BUN, M (Q₁, Q₃) | 4.64 (3.93, 6.07) | 4.64 (3.60,6.07) | 4.64 (3.93,6.07) | 5.00 (3.93,6.07) | 5.00 (3.93,6.40) | 0.093 |
| UA, M (Q₁, Q₃) | 345.00 (285.50, 404.50) | 321.20 (267.70,374.70) | 345.00 (291.50,404.50) | 350.90 (297.40,410.40) | 356.90 (297.40,422.30) | **<.001** |
| eGFR, M (Q₁, Q₃) | 87.10 (64.89, 113.80) | 85.24 (64.07,107.68) | 86.73 (66.29,113.77) | 87.11 (64.48,114.46) | 89.88 (64.88,120.42) | **<.001** |
| ALT, M (Q₁, Q₃) | 22.00 (17.00, 30.75) | 20.00 (16.00,27.00) | 22.00 (17.00,31.00) | 23.00 (17.00,31.00) | 23.00 (17.00,33.00) | **<.001** |
| AST, M (Q₁, Q₃) | 23.00 (19.00, 28.00) | 23.00 (19.00,27.00) | 23.00 (19.00,28.00) | 23.00 (19.00,28.00) | 23.00 (19.00,28.00) | 0.422 |
| ALB, M (Q₁, Q₃) | 42.00 (39.00, 44.00) | 42.00 (40.00,44.00) | 42.00 (40.00,44.00) | 42.00 (39.00,44.00) | 41.00 (38.00,43.00) | **<.001** |
| TBil, M (Q₁, Q₃) | 10.26 (8.55, 13.68) | 11.97 (8.55,15.39) | 10.30 (8.55,13.68) | 10.26 (8.55,13.68) | 10.26 (6.84,12.00) | **<.001** |
| Gender, n (%) |  |  |  |  |  | **<.001** |
| Female | 5,570 (51.89) | 1,295 (48.27) | 1,283 (47.80) | 1,422 (52.98) | 1,570 (58.52) |  |
| Male | 5,164 (48.11) | 1,388 (51.73) | 1,401 (52.20) | 1,262 (47.02) | 1,113 (41.48) |  |
| Race, n (%) |  |  |  |  |  | **<.001** |
| Mexican | 1,966 (18.32) | 286 (10.66) | 507 (18.89) | 568 (21.16) | 605 (22.55) |  |
| Hispanics | 961 (8.95) | 205 (7.64) | 247 (9.20) | 252 (9.39) | 257 (9.58) |  |
| Non-Hispanic White | 4,940 (46.02) | 1,245 (46.40) | 1,206 (44.93) | 1,233 (45.94) | 1,256 (46.81) |  |
| Non-Hispanic Black | 2,067 (19.26) | 676 (25.20) | 497 (18.52) | 460 (17.14) | 434 (16.18) |  |
| Others | 800 (7.45) | 271 (10.10) | 227 (8.46) | 171 (6.37) | 131 (4.88) |  |
| Education level, n (%) |  |  |  |  |  | **<.001** |
| ≤High school | 6,005 (55.94) | 1,339 (49.91) | 1,510 (56.26) | 1,547 (57.64) | 1,609 (59.97) |  |
| College | 2,904 (27.05) | 737 (27.47) | 694 (25.86) | 734 (27.35) | 739 (27.54) |  |
| >College | 1,825 (17) | 607 (22.62) | 480 (17.88) | 403 (15.01) | 335 (12.49) |  |
| Marital status, n (%) |  |  |  |  |  | **<.001** |
| Not married | 4,245 (39.55) | 1,046 (38.99) | 981 (36.55) | 1,070 (39.87) | 1,148 (42.79) |  |
| Married or living with a partner | 6,489 (60.45) | 1,637 (61.01) | 1,703 (63.45) | 1,614 (60.13) | 1,535 (57.21) |  |
| Poverty income ratio, n (%) |  |  |  |  |  | **<.001** |
| <1.3 | 3,145 (29.3) | 649 (24.19) | 708 (26.38) | 841 (31.33) | 947 (35.30) |  |
| 1.3-3.5 | 4,821 (44.91) | 1,168 (43.53) | 1,253 (46.68) | 1,208 (45.01) | 1,192 (44.43) |  |
| >3.5 | 2,768 (25.79) | 866 (32.28) | 723 (26.94) | 635 (23.66) | 544 (20.28) |  |
| BMI, n (%) |  |  |  |  |  | **<.001** |
| <25 | 1,110 (10.34) | 825 (30.75) | 199 (7.41) | 71 (2.65) | 15 (0.56) |  |
| ≥25 and <30 | 3,573 (33.29) | 1,339 (49.91) | 1,268 (47.24) | 732 (27.27) | 234 (8.72) |  |
| ≥30 | 6,051 (56.37) | 519 (19.34) | 1217 (45.34) | 1,881 (70.08) | 2,434 (90.72) |  |
| Smoking status, n (%) |  |  |  |  |  | 0.326 |
| Never | 5,417 (50.47) | 1,404 (52.33) | 1,355 (50.48) | 1,337 (49.81) | 1,321 (49.24) |  |
| Current | 2,015 (18.77) | 499 (18.60) | 506 (18.85) | 504 (18.78) | 506 (18.86) |  |
| Ever | 3,302 (30.76) | 780 (29.07) | 823 (30.66) | 843 (31.41) | 856 (31.90) |  |
| Alcohol use, n (%) |  |  |  |  |  | **<.001** |
| Never | 2,453 (22.85) | 545 (20.31) | 601 (22.39) | 638 (23.77) | 669 (24.93) |  |
| Current | 5,982 (55.73) | 1,611 (60.04) | 1,555 (57.94) | 1,411 (52.57) | 1,405 (52.37) |  |
| Ever | 2,299 (21.42) | 527 (19.64) | 528 (19.67) | 635 (23.66) | 609 (22.70) |  |
| Cancer, n (%) |  |  |  |  |  | 0.998 |
| No | 9,425 (87.81) | 2,358 (87.89) | 2,357 (87.82) | 2,354 (87.70) | 2,356 (87.81) |  |
| Yes | 1,309 (12.19) | 325 (12.11) | 327 (12.18) | 330 (12.30) | 327 (12.19) |  |
| CKD, n (%) |  |  |  |  |  | 0.071 |
| No | 8,536 (79.52) | 2,112 (78.72) | 2,182 (81.30) | 2,121 (79.02) | 2,121 (79.05) |  |
| Yes | 2,198 (20.48) | 571 (21.28) | 502 (18.70) | 563 (20.98) | 562 (20.95) |  |
| CVD, n (%) |  |  |  |  |  | **0.002** |
| No | 9,180 (85.52) | 2,330 (86.84) | 2,322 (86.51) | 2,288 (85.25) | 2,240 (83.49) |  |
| Yes | 1,554 (14.48) | 353 (13.16) | 362 (13.49) | 396 (14.75) | 443 (16.51) |  |
| All-cause  Mortality, n (%) |  |  |  |  |  | 0.085 |
| No | 8,519 (79.36) | 2,165 (80.69) | 2,141 (79.77) | 2,092 (77.94) | 2,121 (79.05) |  |
| Yes | 2,215 (20.64) | 518 (19.31) | 543 (20.23) | 592 (22.06) | 562 (20.95) |  |
| Diabetes Mortality, n (%) |  |  |  |  |  | **<.001** |
| No | 10,374 (96.65) | 2,625 (97.84) | 2,623 (97.73) | 2,589 (96.46) | 2,537 (94.56) |  |
| Yes | 360 (3.35) | 58 (2.16) | 61 (2.27) | 95 (3.54) | 146 (5.44) |  |
| Cardiovascular Mortality, n (%) |  |  |  |  |  | 0.243 |
| No | 10,119 (94.27) | 2,541 (94.71) | 2,541 (94.67) | 2,526 (94.11) | 2,511 (93.59) |  |
| Yes | 615 (5.73) | 142 (5.29) | 143 (5.33) | 158 (5.89) | 172 (6.41) |  |

^*^P-value <0.017 was considered significant, as we had to correct our analysis for multiple testing (P-value of 0.017 was calculated as: 0.05 divided by 3).

Abbreviation: MetS: metabolic syndrome; M: median; Q: quartile; Q_1:_ 1^st^ quartile; Q_3:_ 3^rd^ quartile; n: number; FBG: fasting blood glucose; TyG-WHtR: TyG combining with waist-to-height ratio; TC: total cholesterol; TG: triglyceride; CKD: chronic kidney disease; CVD: cardiovascular disease; eGFR: estimated glomerular filtration rate; BUN: blood urea nitrogen; HDL-C: high-density lipoprotein cholesterol; LDL-C: low-density lipoprotein cholesterol; UA: uric acid; ALT: glutamic-pyruvic transaminase; AST: aspartate transaminase; ALB: albumin; TBil: total bilirubin; BMI: body mass index.

**Table S4**. The Cox regression analysis shows the association between the TyG index with the cause and cause-specific mortality of the MetS population.

| **Subgroup** | **Model 1** | | **Model 2** | | **Model 3** | |
| --- | --- | --- | --- | --- | --- | --- |
|  | **HR (95% CI)** | **P^*^** | **HR (95% CI)** | **P^*^** | **HR (95% CI)** | **P^*^** |
| **All-cause mortality** | | | | | | |
| Q1 | Reference | | Reference | | Reference | |
| Q2 | 1.02(0.90-1.15) | 0.780 | 1.09(0.97-1.23) | 0.160 | 1.10(0.97-1.25) | 0.140 |
| Q3 | 0.99(0.89-1.13) | 0.990 | 1.11(0.98-1.26) | 0.096 | 1.08(0.95-1.23) | 0.232 |
| Q4 | 1.17(1.04-1.31) | **0.011** | 1.45(1.28-1.63) | **<.001** | 1.36(1.18-1.56) | **<.001** |
| **Cardiovascular mortality** | | | | | | |
| Q1 | Reference | | Reference | | Reference | |
| Q2 | 0.96(0.76-1.21) | 0.728 | 1.07(0.84-1.34) | 0.598 | 1.00(0.79-1.27) | 0.998 |
| Q3 | 1.03(0.82-1.30) | 0.778 | 1.20(0.96-1.51) | 0.117 | 1.11(0.87-1.41) | 0.401 |
| Q4 | 1.12(0.90-1.40) | 0.308 | 1.48(1.18-1.87) | **0.001** | 1.29(0.99-1.69) | 0.060 |
| **Diabetes mortality** | | | | | | |
| Q1 | Reference |  | Reference |  | Reference |  |
| Q2 | 1.42(0.98-2.06) | 0.061 | 1.61(1.11-2.33) | **0.012** | 1.52(1.04-2.22) | 0.030 |
| Q3 | 1.47(1.02-2.12) | 0.037 | 1.75(1.21-2.54) | **0.003** | 1.65(1.13-2.40) | **0.010** |
| Q4 | 3.39(2.45-4.68) | **<.001** | 4.38(3.14-6.11) | **<.001** | 4.06(2.81-5.87) | **<.001** |

^*^P-value <0.017 was considered significant, as we had to correct our analysis for multiple testing (P-value of 0.017 was calculated as: 0.05 divided by 3).

Note: Model 1 served as the unadjusted analysis; Model 2: adjusted for age, gender, and race; Model 3: adjusted for age, gender, race, marital status, educational level, family income-poverty ratio, smoking status, alcohol use, cancer, CKD, CVD, BMI, energy intake, serum levels of TC, HDL-C, LDL-C, BUN, UA, eGFR, ALT, AST, ALB, and TBil.

Abbreviation: TyG: triglyceride-glucose; Q: quartile; HR: hazard ratio; CI: confidence interval; MetS: metabolic syndrome; TC: total cholesterol; CKD: chronic kidney disease; CVD: cardiovascular disease; eGFR: estimated glomerular filtration rate; BUN: blood urea nitrogen; HDL-C: high-density lipoprotein cholesterol; LDL-C: low-density lipoprotein cholesterol; UA: uric acid; ALT: glutamic-pyruvic transaminase; AST: aspartate transaminase; ALB: albumin; TBil: total bilirubin; BMI: body mass index.

**Table S5**. The Cox regression analysis shows the association between the TyG-WC index with the cause and cause-specific mortality of the MetS population.

| **Subgroup** | **Model 1** | | **Model 2** | | **Model 3** | |
| --- | --- | --- | --- | --- | --- | --- |
|  | **HR (95% CI)** | **P^*^** | **HR (95% CI)** | **P^*^** | **HR (95% CI)** | **P^*^** |
| **All-cause mortality** | | | | | | |
| Q1 | Reference | | Reference | | Reference | |
| Q2 | 0.93(0.83-1.05) | 0.226 | 1.00(0.89-1.13) | 0.949 | 0.97(0.86-1.10) | 0.645 |
| Q3 | 1.01(0.90-1.13) | 0.893 | 1.14(1.01-1.28) | 0.034 | 1.04(0.92-1.18) | 0.559 |
| Q4 | 1.01(0.90-1.14) | 0.868 | 1.40(1.24-1.58) | **<.001** | 1.17(1.03-1.34) | 0.020 |
| **Cardiovascular mortality** | | | | | | |
| Q1 | Reference | | Reference | | Reference | |
| Q2 | 0.95(0.75-1.19) | 0.632 | 1.03(0.82-1.30) | 0.807 | 1.02(0.81-1.30) | 0.855 |
| Q3 | 1.03(0.82-1.29) | 0.829 | 1.17(0.93-1.47) | 0.187 | 1.09(0.86-1.39) | 0.479 |
| Q4 | 1.21(0.97-1.51) | 0.090 | 1.73(1.37-2.17) | **<.001** | 1.45(1.13-1.85) | **0.004** |
| **Diabetes mortality** | | | | | | |
| Q1 | Reference |  | Reference |  | Reference |  |
| Q2 | 1.12(0.79-1.60) | 0.522 | 1.21(0.85-1.73) | 0.289 | 1.14(0.80-1.63) | 0.480 |
| Q3 | 1.64(1.18-2.27) | **0.003** | 1.85(1.33-2.59) | **<.001** | 1.61(1.15-2.26) | **0.006** |
| Q4 | 2.47(1.81-3.36) | **<.001** | 3.43(2.49-4.72) | **<.001** | 2.55(1.82-3.58) | **<.001** |

^*^P-value <0.017 was considered significant, as we had to correct our analysis for multiple testing (P-value of 0.017 was calculated as: 0.05 divided by 3).

Note: Model 1 served as the unadjusted analysis; Model 2: adjusted for age, gender, and race; Model 3: adjusted for age, gender, race, marital status, educational level, family income-poverty ratio, smoking status, alcohol use, cancer, CKD, CVD, energy intake, serum levels of TC, HDL-C, LDL-C, BUN, UA, eGFR, ALT, AST, ALB, and TBil.

Abbreviation: TyG: triglyceride-glucose; TyG-WC: TyG combining with waist circumference; Q: quartile; HR: hazard ratio; CI: confidence interval; MetS: metabolic syndrome; TC: total cholesterol; CKD: chronic kidney disease; CVD: cardiovascular disease; eGFR: estimated glomerular filtration rate; BUN: blood urea nitrogen; HDL-C: high-density lipoprotein cholesterol; LDL-C: low-density lipoprotein cholesterol; UA: uric acid; ALT: glutamic-pyruvic transaminase; AST: aspartate transaminase; ALB: albumin; TBil: total bilirubin.

**Table S6**. The Cox regression analysis shows the association between the TyG-WHtR index with the cause and cause-specific mortality of the MetS population.

| **Subgroup** | **Model 1** | | **Model 2** | | **Model 3** | |
| --- | --- | --- | --- | --- | --- | --- |
|  | **HR (95% CI)** | **P^*^** | **HR (95% CI)** | **P^*^** | **HR (95% CI)** | **P^*^** |
| **All-cause mortality** | | | | | | |
| Q1 | Reference | | Reference | | Reference | |
| Q2 | 1.01(0.90-1.14) | 0.878 | 1.07(0.95-1.20) | 0.297 | 1.02(0.90-1.15) | 0.816 |
| Q3 | 1.14(1.02-1.29) | 0.027 | 1.21(1.07-1.36) | **0.002** | 1.06(0.94-1.20) | 0.340 |
| Q4 | 1.16(1.03-1.30) | 0.017 | 1.54(1.37-1.74) | **<.001** | 1.29(1.13-1.47) | **<.001** |
| **Cardiovascular mortality** | | | | | | |
| Q1 | Reference | | Reference | | Reference | |
| Q2 | 0.97(0.77-1.22) | 0.801 | 1.05(0.83-1.32) | 0.689 | 1.00(0.79-1.27) | 0.989 |
| Q3 | 1.11(0.89-1.40) | 0.357 | 1.22(0.97-1.53) | 0.091 | 1.07(0.85-1.36) | 0.564 |
| Q4 | 1.29(1.03-1.61) | 0.026 | 1.83(1.46-2.30) | **<.001** | 1.50(1.17-1.92) | **0.002** |
| **Diabetes mortality** | | | | | | |
| Q1 | Reference |  | Reference |  | Reference |  |
| Q2 | 1.01(0.71-1.45) | 0.949 | 1.07(0.74-1.53) | 0.729 | 0.96(0.67-1.39) | 0.845 |
| Q3 | 1.64(1.18-2.27) | **0.003** | 1.71(1.23-2.38) | **0.001** | 1.41(1.01-1.98) | 0.046 |
| Q4 | 2.69(1.98-3.64) | **<.001** | 3.45(2.53-4.71) | **<.001** | 2.53(1.81-3.54) | **<.001** |

^*^P-value <0.017 was considered significant, as we had to correct our analysis for multiple testing (P-value of 0.017 was calculated as: 0.05 divided by 3).

Note: Model 1 served as the unadjusted analysis; Model 2: adjusted for age, gender, and race; Model 3: adjusted for age, gender, race, marital status, educational level, family income-poverty ratio, smoking status, alcohol use, cancer, CKD, CVD, energy intake, serum levels of TC, HDL-C, LDL-C, BUN, UA, eGFR, ALT, AST, ALB, and TBil.

Abbreviation: TyG: triglyceride-glucose; TyG-WHtR: TyG combining with waist circumference to height ratio; Q: quartile; HR: hazard ratio; CI: confidence interval; MetS: metabolic syndrome; TC: total cholesterol; CKD: chronic kidney disease; CVD: cardiovascular disease; eGFR: estimated glomerular filtration rate; BUN: blood urea nitrogen; HDL-C: high-density lipoprotein cholesterol; LDL-C: low-density lipoprotein cholesterol; UA: uric acid; ALT: glutamic-pyruvic transaminase; AST: aspartate transaminase; ALB: albumin; TBil: total bilirubin.

**Table S7**. Sensitive analysis shows the robust association between the TyG index with the cause and cause-specific mortality of the MetS population. (Excluding the participants with diabetes mellitus at baseline).

| **Subgroup** | **Model 1** | | **Model 2** | | **Model 3** | |
| --- | --- | --- | --- | --- | --- | --- |
|  | **HR (95% CI)** | **P^*^** | **HR (95% CI)** | **P^*^** | **HR (95% CI)** | **P^*^** |
| **All-cause mortality** | | | | | | |
| Q1 | Reference | | Reference | | Reference | |
| Q2 | 1.03(0.90-1.18) | 0.700 | 1.09(0.95-1.25) | 0.229 | 1.09(0.94-1.26) | 0.248 |
| Q3 | 0.81(0.98-1.13) | 0.812 | 1.08(0.94-1.25) | 0.295 | 1.08(0.93-1.27) | 0.317 |
| Q4 | 0.96(0.83-1.12) | 0.598 | 1.22(1.05-1.43) | **0.011** | 1.24(1.03-1.50) | 0.027 |
| **Cardiovascular mortality** | | | | | | |
| Q1 | Reference | | Reference | | Reference | |
| Q2 | 1.02(0.78-1.33) | 0.904 | 1.12(0.85-1.46) | 0.423 | 1.01(0.77-1.34) | 0.921 |
| Q3 | 1.02(0.78-1.34) | 0.870 | 1.18(0.90-1.55) | 0.244 | 1.09(0.81-1.47) | 0.557 |
| Q4 | 0.82(0.60-1.10) | 0.184 | 1.10(0.81-1.50) | 0.549 | 0.98(0.67-1.42) | 0.903 |
| **Diabetes mortality** | | | |  |  |  |
| Q1 | Reference |  | Reference |  | Reference |  |
| Q2 | 1.98(1.03-3.83) | 0.041 | 2.28(1.18-4.43) | **0.015** | 2.03(1.03-4.00) | 0.042 |
| Q3 | 2.36(1.24-4.50) | **0.009** | 2.92(1.51-5.64) | **0.001** | 2.47(1.24-4.94) | **0.010** |
| Q4 | 4.12(2.22-7.63) | **<.001** | 5.58(2.95-10.53) | **<.001** | 4.92(2.36-10.26) | **<.001** |

^*^P-value <0.017 was considered significant, as we had to correct our analysis for multiple testing (P-value of 0.017 was calculated as: 0.05 divided by 3).

Note: Model 1 served as the unadjusted analysis; Model 2: adjusted for age, gender, and race; Model 3: adjusted for age, gender, race, marital status, educational level, family income-poverty ratio, smoking status, alcohol use, cancer, CKD, CVD, BMI, energy intake, serum levels of TC, HDL-C, LDL-C, BUN, UA, eGFR, ALT, AST, ALB, and TBil.

Abbreviation: TyG: triglyceride-glucose; Q: quartile; HR: hazard ratio; CI: confidence interval; MetS: metabolic syndrome; TC: total cholesterol; CKD: chronic kidney disease; CVD: cardiovascular disease; eGFR: estimated glomerular filtration rate; BUN: blood urea nitrogen; HDL-C: high-density lipoprotein cholesterol; LDL-C: low-density lipoprotein cholesterol; UA: uric acid; ALT: glutamic-pyruvic transaminase; AST: aspartate transaminase; ALB: albumin; TBil: total bilirubin; BMI: body mass index.

**Table S8**. Sensitive analysis shows the robust association between the TyG-WC index with the cause and cause-specific mortality of the MetS population. (Excluding the participants with diabetes mellitus at baseline).

| **Subgroup** | **Model 1** | | **Model 2** | | **Model 3** | |
| --- | --- | --- | --- | --- | --- | --- |
|  | **HR (95% CI)** | **P^*^** | **HR (95% CI)** | **P^*^** | **HR (95% CI)** | **P^*^** |
| **All-cause mortality** | | | | | | |
| Q1 | Reference | | Reference | | Reference | |
| Q2 | 0.87(0.75-0.99) | 0.041 | 0.98(0.85-1.13) | 0.779 | 0.94(0.81-1.08) | 0.363 |
| Q3 | 0.93(0.81-1.07) | 0.305 | 1.10(0.95-1.26) | 0.212 | 0.97(0.84-1.13) | 0.690 |
| Q4 | 0.85(0.74-0.99) | 0.037 | 1.29(1.10-1.51) | **0.001** | 1.09(0.92-1.29) | 0.328 |
| **Cardiovascular mortality** | | | | | | |
| Q1 | Reference | | Reference | | Reference | |
| Q2 | 0.90(0.69-1.18) | 0.434 | 1.03(0.78-1.35) | 0.859 | 1.02(0.77-1.35) | 0.890 |
| Q3 | 0.97(0.74-1.26) | 0.794 | 1.13(0.86-1.50) | 0.378 | 1.04(0.78-1.39) | 0.802 |
| Q4 | 0.99(0.75-1.31) | 0.943 | 1.51(1.13-2.03) | **0.006** | 1.28(0.93-1.76) | 0.135 |
| **Diabetes mortality** | | | | | |  |
| Q1 | Reference |  | Reference |  | Reference |  |
| Q2 | 1.12(0.63-1.98) | 0.706 | 1.26(0.71-2.25) | 0.431 | 1.10(0.61-1.98) | 0.757 |
| Q3 | 1.33(0.76-2.32) | 0.320 | 1.53(0.86-2.71) | 0.149 | 1.20(0.66-2.17) | 0.545 |
| Q4 | 2.52(1.51-4.21) | **<.001** | 3.76(2.20-6.42) | **<.001** | 2.72(1.53-4.84) | **0.001** |

^*^P-value <0.017 was considered significant, as we had to correct our analysis for multiple testing (P-value of 0.017 was calculated as: 0.05 divided by 3).

Note: Model 1 served as the unadjusted analysis; Model 2: adjusted for age, gender, and race; Model 3: adjusted for age, gender, race, marital status, educational level, family income-poverty ratio, smoking status, alcohol use, cancer, CKD, CVD, energy intake, serum levels of TC, HDL-C, LDL-C, BUN, UA, eGFR, ALT, AST, ALB, and TBil.

Abbreviation: TyG: triglyceride-glucose; TyG-WC: TyG combining with waist circumference; Q: quartile; HR: hazard ratio; CI: confidence interval; MetS: metabolic syndrome; TC: total cholesterol; CKD: chronic kidney disease; CVD: cardiovascular disease; eGFR: estimated glomerular filtration rate; BUN: blood urea nitrogen; HDL-C: high-density lipoprotein cholesterol; LDL-C: low-density lipoprotein cholesterol; UA: uric acid; ALT: glutamic-pyruvic transaminase; AST: aspartate transaminase; ALB: albumin; TBil: total bilirubin.

**Table S9**. Sensitive analysis shows the robust association between the TyG-WHtR index with the cause and cause-specific mortality of the MetS population. (Excluding the participants with diabetes mellitus at baseline).

| **Subgroup** | **Model 1** | | **Model 2** | | **Model 3** | |
| --- | --- | --- | --- | --- | --- | --- |
|  | **HR (95% CI)** | **P^*^** | **HR (95% CI)** | **P^*^** | **HR (95% CI)** | **P^*^** |
| **All-cause mortality** | | | | | | |
| Q1 | Reference | | Reference | | Reference | |
| Q2 | 0.95(0.83-1.10) | 0.496 | 1.03(0.89-1.18) | 0.712 | 0.96(0.83-1.11) | 0.596 |
| Q3 | 1.10(0.96-1.26) | 0.189 | 1.17(1.02-1.35) | 0.027 | 1.02(0.88-1.18) | 0.847 |
| Q4 | 1.04(0.89-1.21) | 0.634 | 1.46(1.25-1.70) | **<.001** | 1.20(1.02-1.43) | 0.033 |
| **Cardiovascular mortality** | | | | | | |
| Q1 | Reference | | Reference | | Reference | |
| Q2 | 0.89(0.67-1.16) | 0.380 | 0.98(0.74-1.29) | 0.884 | 0.90(0.68-1.20) | 0.477 |
| Q3 | 1.10(0.84-1.44) | 0.477 | 1.21(0.93-1.58) | 0.163 | 1.04(0.79-1.39) | 0.769 |
| Q4 | 1.06(0.79-1.41) | 0.704 | 1.58(1.18-2.11) | **0.002** | 1.24(0.89-1.71) | 0.200 |
| **Diabetes mortality** | |  |  |  |  |  |
| Q1 | Reference |  | Reference |  | Reference |  |
| Q2 | 0.84(0.46-1.56) | 0.588 | 0.91(0.49-1.69) | 0.771 | 0.74(0.39-1.38) | 0.339 |
| Q3 | 1.73(1.01-2.94) | 0.045 | 1.82(1.06-3.13) | 0.031 | 1.34(0.76-2.35) | 0.309 |
| Q4 | 2.69(1.61-4.51) | **<.001** | 3.67(2.16-6.25) | **<.001** | 2.51(1.40-4.47) | **0.002** |

^*^P-value <0.017 was considered significant, as we had to correct our analysis for multiple testing (P-value of 0.017 was calculated as: 0.05 divided by 3).

Note: Model 1 served as the unadjusted analysis; Model 2: adjusted for age, gender, and race; Model 3: adjusted for age, gender, race, marital status, educational level, family income-poverty ratio, smoking status, alcohol use, cancer, CKD, CVD, energy intake, serum levels of TC, HDL-C, LDL-C, BUN, UA, eGFR, ALT, AST, ALB, and TBil.

Abbreviation: TyG: triglyceride-glucose; TyG-WHtR: TyG combining with waist circumference to height ratio; Q: quartile; HR: hazard ratio; CI: confidence interval; MetS: metabolic syndrome; TC: total cholesterol; CKD: chronic kidney disease; CVD: cardiovascular disease; eGFR: estimated glomerular filtration rate; BUN: blood urea nitrogen; HDL-C: high-density lipoprotein cholesterol; LDL-C: low-density lipoprotein cholesterol; UA: uric acid; ALT: glutamic-pyruvic transaminase; AST: aspartate transaminase; ALB: albumin; TBil: total bilirubin.

**Table S10**. Sensitive analysis shows the robust association between the TyG index with the cause and cause-specific mortality of the MetS population. (Excluding the participants who died within 2 years).

| **Subgroup** | **Model 1** | | **Model 2** | | **Model 3** | |
| --- | --- | --- | --- | --- | --- | --- |
|  | **HR (95% CI)** | **P^*^** | **HR (95% CI)** | **P^*^** | **HR (95% CI)** | **P^*^** |
| **All-cause mortality** | | | | | | |
| Q1 | Reference | | Reference | | Reference | |
| Q2 | 1.02(0.90-1.16) | 0.740 | 1.10(0.96-1.25) | 0.167 | 1.08(0.95-1.24) | 0.257 |
| Q3 | 0.98(0.86-1.12) | 0.765 | 1.09(0.95-1.24) | 0.207 | 1.03(0.90-1.18) | 0.691 |
| Q4 | 1.16(1.02-1.31) | 0.025 | 1.44(1.26-1.63) | **<.001** | 1.32(1.13-1.53) | **<.001** |
| **Cardiovascular mortality** | | | | | | |
| Q1 | Reference | | Reference | | Reference | |
| Q2 | 0.95(0.74-1.22) | 0.710 | 1.07(0.83-1.37) | 0.599 | 1.01(0.78-1.30) | 0.968 |
| Q3 | 1.02(0.80-1.29) | 0.904 | 1.20(0.94-1.53) | 0.153 | 1.10(0.85-1.43) | 0.458 |
| Q4 | 1.10(0.87-1.40) | 0.427 | 1.48(1.16-1.89) | **0.002** | 1.32(0.99-1.77) | 0.058 |
| **Diabetes mortality** | | | | | | |
| Q1 | Reference |  | Reference |  | Reference |  |
| Q2 | 1.59(1.06-2.39) | 0.025 | 1.80(1.20-2.71) | **0.005** | 1.62(1.06-2.46) | 0.025 |
| Q3 | 1.63(1.09-2.44) | 0.018 | 1.95(1.29-2.92) | **0.001** | 1.79(1.18-2.73) | **0.006** |
| Q4 | 3.81(2.66-5.46) | **<.001** | 4.94(3.42-7.15) | **<.001** | 4.62(3.07-6.95) | **<.001** |

^*^P-value <0.017 was considered significant, as we had to correct our analysis for multiple testing (P-value of 0.017 was calculated as: 0.05 divided by 3).

Note: Model 1 served as the unadjusted analysis; Model 2: adjusted for age, gender, and race; Model 3: adjusted for age, gender, race, marital status, educational level, family income-poverty ratio, smoking status, alcohol use, cancer, CKD, CVD, BMI, energy intake, serum levels of TC, HDL-C, LDL-C, BUN, UA, eGFR, ALT, AST, ALB, and TBil.

Abbreviation: TyG: triglyceride-glucose; Q: quartile; HR: hazard ratio; CI: confidence interval; MetS: metabolic syndrome; TC: total cholesterol; CKD: chronic kidney disease; CVD: cardiovascular disease; eGFR: estimated glomerular filtration rate; BUN: blood urea nitrogen; HDL-C: high-density lipoprotein cholesterol; LDL-C: low-density lipoprotein cholesterol; UA: uric acid; ALT: glutamic-pyruvic transaminase; AST: aspartate transaminase; ALB: albumin; TBil: total bilirubin; BMI: body mass index.

**Table S11**. Sensitive analysis shows the robust association between the TyG-WC index with the cause and cause-specific mortality of the MetS population. (Excluding the participants who died within 2 years).

| **Subgroup** | **Model 1** | | **Model 2** | | **Model 3** | |
| --- | --- | --- | --- | --- | --- | --- |
|  | **HR (95% CI)** | **P^*^** | **HR (95% CI)** | **P^*^** | **HR (95% CI)** | **P^*^** |
| **All-cause mortality** | | | | | | |
| Q1 | Reference | | Reference | | Reference | |
| Q2 | 0.90(0.79-1.02) | 0.096 | 0.97(0.86-1.11) | 0.685 | 0.95(0.83-1.08) | 0.398 |
| Q3 | 1.02(0.90-1.16) | 0.740 | 1.17(1.03-1.32) | 0.018 | 1.07(0.93-1.22) | 0.345 |
| Q4 | 1.00(0.88-1.14) | 0.995 | 1.39(1.22-1.59) | **<.001** | 1.19(1.03-1.37) | 0.019 |
| **Cardiovascular mortality** | | | | | | |
| Q1 | Reference | | Reference | | Reference | |
| Q2 | 0.91(0.71-1.17) | 0.442 | 0.99(0.77-1.28) | 0.937 | 1.10(0.77-1.29) | 0.969 |
| Q3 | 1.05(0.82-1.33) | 0.722 | 1.20(0.94-1.54) | 0.152 | 1.14(0.88-1.47) | 0.331 |
| Q4 | 1.23(0.97-1.56) | 0.088 | 1.75(1.37-2.25) | **<.001** | 1.52(1.16-1.98) | **0.002** |
| **Diabetes mortality** | | | | | | |
| Q1 | Reference |  | Reference |  | Reference |  |
| Q2 | 1.17(0.80-1.72) | 0.422 | 1.27(0.86-1.87) | 0.229 | 1.19(0.81-1.76) | 0.375 |
| Q3 | 1.86(1.31-2.64) | **0.001** | 2.10(1.47-3.01) | **<.001** | 1.85(1.29-2.67) | **0.001** |
| Q4 | 2.57(1.83-3.61) | **<.001** | 3.55(2.50-5.04) | **<.001** | 2.68(1.86-3.87) | **<.001** |

^*^P-value <0.017 was considered significant, as we had to correct our analysis for multiple testing (P-value of 0.017 was calculated as: 0.05 divided by 3).

Note: Model 1 served as the unadjusted analysis; Model 2: adjusted for age, gender, and race; Model 3: adjusted for age, gender, race, marital status, educational level, family income-poverty ratio, smoking status, alcohol use, cancer, CKD, CVD, energy intake, serum levels of TC, HDL-C, LDL-C, BUN, UA, eGFR, ALT, AST, ALB, and TBil.

Abbreviation: TyG: triglyceride-glucose; TyG-WC: TyG combining with waist circumference; Q: quartile; HR: hazard ratio; CI: confidence interval; MetS: metabolic syndrome; TC: total cholesterol; CKD: chronic kidney disease; CVD: cardiovascular disease; eGFR: estimated glomerular filtration rate; BUN: blood urea nitrogen; HDL-C: high-density lipoprotein cholesterol; LDL-C: low-density lipoprotein cholesterol; UA: uric acid; ALT: glutamic-pyruvic transaminase; AST: aspartate transaminase; ALB: albumin; TBil: total bilirubin.

**Table S12**. Sensitive analysis shows the robust association between the TyG-WHtR index with the cause and cause-specific mortality of the MetS population. (Excluding the participants who died within 2 years).

| **Subgroup** | **Model 1** | | **Model 2** | | **Model 3** | |
| --- | --- | --- | --- | --- | --- | --- |
|  | **HR (95% CI)** | **P^*^** | **HR (95% CI)** | **P^*^** | **HR (95% CI)** | **P^*^** |
| **All-cause mortality** | | | | | | |
| Q1 | Reference | | Reference | | Reference | |
| Q2 | 1.00(0.88-1.14) | 0.995 | 1.06(0.93-1.21) | 0.393 | 1.01(0.89-1.15) | 0.875 |
| Q3 | 1.15(1.01-1.31) | 0.030 | 1.22(1.07-1.38) | **0.003** | 1.08(0.94-1.23) | 0.285 |
| Q4 | 1.15(1.01-1.31) | 0.033 | 1.53(1.35-1.75) | **<.001** | 1.30(1.13-1.50) | **<.001** |
| **Cardiovascular mortality** | | | | | | |
| Q1 | Reference | | Reference | | Reference | |
| Q2 | 0.98(0.77-1.26) | 0.886 | 1.07(0.83-1.37) | 0.591 | 1.03(0.80-1.33) | 0.819 |
| Q3 | 1.10(0.86-1.40) | 0.455 | 1.21(0.95-1.55) | 0.128 | 1.08(0.84-1.40) | 0.547 |
| Q4 | 1.28(1.01-1.63) | 0.041 | 1.84(1.44-2.35) | **<.001** | 1.55(1.19-2.03) | **0.001** |
| **Diabetes mortality** | | | | | | |
| Q1 | Reference |  | Reference |  | Reference |  |
| Q2 | 1.08(0.73-1.59) | 0.708 | 1.14(0.77-1.68) | 0.518 | 1.02(0.69-1.52) | 0.911 |
| Q3 | 1.83(1.29-2.60) | **0.001** | 1.91(1.34-2.72) | **<.001** | 1.57(1.09-2.27) | **0.015** |
| Q4 | 2.81(2.01-3.91) | **<.001** | 3.56(2.54-5.01) | **<.001** | 2.64(1.83-3.80) | **<.001** |

^*^P-value <0.017 was considered significant, as we had to correct our analysis for multiple testing (P-value of 0.017 was calculated as: 0.05 divided by 3).

Note: Model 1 served as the unadjusted analysis; Model 2: adjusted for age, gender, and race; Model 3: adjusted for age, gender, race, marital status, educational level, family income-poverty ratio, smoking status, alcohol use, cancer, CKD, CVD, energy intake, serum levels of TC, HDL-C, LDL-C, BUN, UA, eGFR, ALT, AST, ALB, and TBil.

Abbreviation: TyG: triglyceride-glucose; TyG-WHtR: TyG combining with waist circumference to height ratio; Q: quartile; HR: hazard ratio; CI: confidence interval; MetS: metabolic syndrome; TC: total cholesterol; CKD: chronic kidney disease; CVD: cardiovascular disease; eGFR: estimated glomerular filtration rate, BUN: blood urea nitrogen, HDL-C: high-density lipoprotein cholesterol, LDL-C: low-density lipoprotein cholesterol; UA: uric acid; ALT: glutamic-pyruvic transaminase; AST: aspartate transaminase; ALB: albumin; TBil: total bilirubin.

**Table S13**. Sensitive analysis shows the robust association between the TyG index with the cause and cause-specific mortality of the MetS population. (Excluding the participants with a history of comorbidities).

| **Subgroup** | **Model 1** | | **Model 2** | | **Model 3** | |
| --- | --- | --- | --- | --- | --- | --- |
|  | **HR (95% CI)** | **P^*^** | **HR (95% CI)** | **P^*^** | **HR (95% CI)** | **P^*^** |
| **All-cause mortality** | | | | | | |
| Q1 | Reference | | Reference | | Reference | |
| Q2 | 1.09(0.89-1.34) | 0.422 | 1.20(0.97-1.48) | 0.089 | 1.27(1.02-1.57) | 0.032 |
| Q3 | 1.10(0.90-1.35) | 0.360 | 1.21(0.99-1.50) | 0.069 | 1.20(0.96-1.49) | 0.115 |
| Q4 | 1.39(1.15-1.70) | **0.001** | 1.69(1.38-2.08) | **<.001** | 1.67(1.31-2.13) | **<.001** |
| **Cardiovascular mortality** | | | | | | |
| Q1 | Reference | | Reference | | Reference | |
| Q2 | 1.19(0.80-1.79) | 0.393 | 1.39(0.92-2.08) | 0.116 | 1.48(0.97-2.25) | 0.066 |
| Q3 | 0.94(0.62-1.45) | 0.792 | 1.11(0.72-1.71) | 0.633 | 1.08(0.69-1.70) | 0.737 |
| Q4 | 1.32(0.89-1.97) | 0.166 | 1.74(1.16-2.63) | **0.008** | 1.67(1.03-2.70) | 0.037 |
| **Diabetes mortality** | | | | | | |
| Q1 | Reference |  | Reference |  | Reference |  |
| Q2 | 1.55(0.84-2.89) | 0.162 | 1.91(1.02-3.56) | 0.043 | 1.93(1.02-3.66) | 0.045 |
| Q3 | 1.37(0.73-2.58) | 0.331 | 1.77(0.93-3.36) | 0.084 | 1.67(0.86-3.25) | 0.133 |
| Q4 | 3.38(1.95-5.89) | **<.001** | 4.63(2.60-8.24) | **<.001** | 4.91(2.56-9.42) | **<.001** |

^*^P-value <0.017 was considered significant, as we had to correct our analysis for multiple testing (P-value of 0.017 was calculated as: 0.05 divided by 3).

Note: Model 1 served as the unadjusted analysis; Model 2: adjusted for age, gender, and race; Model 3: adjusted for age, gender, race, marital status, educational level, family income-poverty ratio, smoking status, alcohol use, BMI, energy intake, serum levels of TC, HDL-C, LDL-C, BUN, UA, eGFR, ALT, AST, ALB, and TBil.

Abbreviation: TyG: triglyceride-glucose; Q: quartile; HR: hazard ratio; CI: confidence interval; MetS: metabolic syndrome; TC: total cholesterol; eGFR: estimated glomerular filtration rate; BUN: blood urea nitrogen; HDL-C: high-density lipoprotein cholesterol; LDL-C: low-density lipoprotein cholesterol; UA: uric acid; ALT: glutamic-pyruvic transaminase; AST: aspartate transaminase; ALB: albumin; TBil: total bilirubin; BMI: body mass index.

**Table S14**. Sensitive analysis shows the robust association between the TyG-WC index with the cause and cause-specific mortality of the MetS population. (Excluding the participants with a history of comorbidities).

| **Subgroup** | **Model 1** | | **Model 2** | | **Model 3** | |
| --- | --- | --- | --- | --- | --- | --- |
|  | **HR (95% CI)** | **P** | **HR (95% CI)** | **P** | **HR (95% CI)** | **P** |
| **All-cause mortality** | | | | | | |
| Q1 | Reference | | Reference | | Reference | |
| Q2 | 0.89(0.73-1.08) | 0.234 | 0.94(0.77-1.15) | 0.528 | 0.92(0.75-1.12) | 0.399 |
| Q3 | 0.95(0.79-1.15) | 0.601 | 1.06(0.87-1.29) | 0.579 | 1.00(0.81-1.23) | 0.983 |
| Q4 | 1.02(0.84-1.24) | 0.852 | 1.35(1.10-1.66) | **0.004** | 1.22(0.97-1.52) | 0.090 |
| **Cardiovascular mortality** | | | | | | |
| Q1 | Reference | | Reference | | Reference | |
| Q2 | 1.02(0.68-1.53) | 0.919 | 1.06(0.70-1.60) | 0.797 | 1.09(0.71-1.66) | 0.693 |
| Q3 | 1.08(0.73-1.61) | 0.702 | 1.19(0.79-1.79) | 0.409 | 1.22(0.79-1.88) | 0.364 |
| Q4 | 1.27(0.85-1.88) | 0.247 | 1.67(1.10-2.53) | **0.017** | 1.62(1.03-2.57) | **0.039** |
| **Diabetes mortality** | | | | | | |
| Q1 | Reference |  | Reference |  | Reference |  |
| Q2 | 0.87(0.48-1.58) | 0.649 | 0.97(0.53-1.76) | 0.916 | 0.86(0.47-1.58) | 0.624 |
| Q3 | 1.41(0.83-2.39) | 0.200 | 1.64(0.96-2.82) | 0.071 | 1.49(0.85-2.62) | 0.163 |
| Q4 | 2.14(1.30-3.53) | **0.003** | 3.06(1.81-5.17) | **<.001** | 2.50(1.42-4.41) | **0.002** |

Note: Model 1 served as the unadjusted analysis; Model 2: adjusted for age, gender, and race; Model 3: adjusted for age, gender, race, marital status, educational level, family income-poverty ratio, smoking status, alcohol use, energy intake, serum levels of TC, HDL-C, LDL-C, BUN, UA, eGFR, ALT, AST, ALB, and TBil.

Abbreviation: TyG: triglyceride-glucose; TyG-WC: TyG combining with waist circumference; Q: quartile; HR: hazard ratio; CI: confidence interval; MetS: metabolic syndrome; TC: total cholesterol; eGFR: estimated glomerular filtration rate; BUN: blood urea nitrogen; HDL-C: high-density lipoprotein cholesterol; LDL-C: low-density lipoprotein cholesterol; UA: uric acid; ALT: glutamic-pyruvic transaminase; AST: aspartate transaminase; ALB: albumin; TBil: total bilirubin.

**Table S15**. Sensitive analysis shows the robust association between the TyG-WHtR index with the cause and cause-specific mortality of the MetS population. (Excluding the participants with a history of comorbidities).

| **Subgroup** | **Model 1** | | **Model 2** | | **Model 3** | |
| --- | --- | --- | --- | --- | --- | --- |
|  | **HR (95% CI)** | **P^*^** | **HR (95% CI)** | **P^*^** | **HR (95% CI)** | **P^*^** |
| **All-cause mortality** | | | | | | |
| Q1 | Reference | | Reference | | Reference | |
| Q2 | 1.09(0.89-1.33) | 0.397 | 1.14(0.93-1.39) | 0.215 | 1.08(0.88-1.33) | 0.482 |
| Q3 | 1.22(1.00-1.49) | **0.048** | 1.22(0.99-1.49) | 0.054 | 1.16(0.94-1.44) | 0.164 |
| Q4 | 1.31(1.07-1.60) | **0.008** | 1.61(1.32-1.98) | **<.001** | 1.43(1.14-1.79) | **0.002** |
| **Cardiovascular mortality** | | | | | | |
| Q1 | Reference | | Reference | | Reference | |
| Q2 | 0.90(0.59-1.36) | 0.602 | 0.95(0.62-1.44) | 0.799 | 0.91(0.59-1.40) | 0.657 |
| Q3 | 1.29(0.88-1.89) | 0.199 | 1.34(0.90-1.97) | 0.146 | 1.30(0.86-1.97) | 0.218 |
| Q4 | 1.22(0.81-1.81) | 0.340 | 1.60(1.06-2.40) | **0.025** | 1.44(0.92-2.28) | 0.114 |
| **Diabetes mortality** | | | | | | |
| Q1 | Reference |  | Reference |  | Reference |  |
| Q2 | 1.05(0.58-1.89) | 0.878 | 1.10(0.61-1.99) | 0.760 | 0.86(0.47-1.59) | 0.635 |
| Q3 | 1.33(0.76-2.35) | 0.320 | 1.37(0.77-2.43) | 0.279 | 1.11(0.61-2.00) | 0.738 |
| Q4 | 2.82(1.70-4.67) | **<.001** | 3.54(2.11-5.95) | **<.001** | 2.71(1.53-4.79) | **0.001** |

^*^P-value <0.017 was considered significant, as we had to correct our analysis for multiple testing (P-value of 0.017 was calculated as: 0.05 divided by 3).

Note: Model 1 served as the unadjusted analysis; Model 2: adjusted for age, gender, and race; Model 3: adjusted for age, gender, race, marital status, educational level, family income-poverty ratio, smoking status, alcohol use, energy intake, serum levels of TC, HDL-C, LDL-C, BUN, UA, eGFR, ALT, AST, ALB, and TBil.

Abbreviation: TyG: triglyceride-glucose; TyG-WHtR: TyG combining with waist circumference to height ratio; Q: quartile; HR: hazard ratio; CI: confidence interval; MetS: metabolic syndrome; TC: total cholesterol; eGFR: estimated glomerular filtration rate; BUN: blood urea nitrogen; HDL-C: high-density lipoprotein cholesterol; LDL-C: low-density lipoprotein cholesterol; UA: uric acid; ALT: glutamic-pyruvic transaminase; AST: aspartate transaminase; ALB: albumin; TBil: total bilirubin.

**Table S16**. Sensitive analysis shows the robust association between the TyG index with the cause and cause-specific mortality of the MetS population. (Excluding the participants with low or normal BMI at baseline).

| **Subgroup** | **Model 1** | | **Model 2** | | **Model 3** | |
| --- | --- | --- | --- | --- | --- | --- |
|  | **HR (95% CI)** | **P^*^** | **HR (95% CI)** | **P^*^** | **HR (95% CI)** | **P^*^** |
| **All-cause mortality** | | | | | | |
| Q1 | Reference | | Reference | | Reference | |
| Q2 | 1.07(0.94-1.22) | 0.326 | 1.13(0.99-1.29) | 0.073 | 1.10(0.96-1.26) | 0.180 |
| Q3 | 1.02(0.89-1.17) | 0.771 | 1.10(0.96-1.26) | 0.173 | 1.04(0.90-1.20) | 0.614 |
| Q4 | 1.25(1.10-1.42) | **0.001** | 1.46(1.28-1.67) | **<.001** | 1.30(1.11-1.51) | **0.001** |
| **Cardiovascular mortality** | | | | | | |
| Q1 | Reference | | Reference | | Reference | |
| Q2 | 1.00(0.78-1.28) | 0.970 | 1.10(0.86-1.41) | 0.458 | 0.99(0.77-1.29) | 0.957 |
| Q3 | 1.01(0.79-1.29) | 0.951 | 1.15(0.90-1.48) | 0.262 | 1.03(0.79-1.34) | 0.814 |
| Q4 | 1.14(0.90-1.45) | 0.281 | 1.45(1.13-1.86) | **0.003** | 1.22(0.91-1.63) | 0.182 |
| **Diabetes mortality** | | | | | |  |
| Q1 | Reference |  | Reference |  | Reference |  |
| Q2 | 1.53(1.04-2.27) | 0.033 | 1.70(1.15-2.53) | **0.008** | 1.47(0.99-2.20) | 0.059 |
| Q3 | 1.37(0.92-2.04) | 0.123 | 1.59(1.06-2.38) | 0.025 | 1.52(1.02-2.28) | 0.042 |
| Q4 | 3.44(2.43-4.88) | **<.001** | 4.27(2.98-6.12) | **<.001** | 3.62(2.44-5.38) | **<.001** |

^*^P-value <0.017 was considered significant, as we had to correct our analysis for multiple testing (P-value of 0.017 was calculated as: 0.05 divided by 3).

Note: Model 1 served as the unadjusted analysis; Model 2: adjusted for age, gender, and race; Model 3: adjusted for age, gender, race, marital status, educational level, family income-poverty ratio, smoking status, alcohol use, cancer, CKD, CVD, BMI, energy intake, serum levels of TC, HDL-C, LDL-C, BUN, UA, eGFR, ALT, AST, ALB, and TBil.

Abbreviation: TyG: triglyceride-glucose; Q: quartile; HR: hazard ratio; CI: confidence interval; MetS: metabolic syndrome; TC: total cholesterol; CKD: chronic kidney disease; CVD: cardiovascular disease; eGFR: estimated glomerular filtration rate; BUN: blood urea nitrogen; HDL-C: high-density lipoprotein cholesterol; LDL-C: low-density lipoprotein cholesterol; UA: uric acid; ALT: glutamic-pyruvic transaminase; AST: aspartate transaminase; ALB: albumin; TBil: total bilirubin; BMI: body mass index.

**Table S17**. Sensitive analysis shows the robust association between the TyG-WC index with the cause and cause-specific mortality of the MetS population. (Excluding the participants with low or normal BMI at baseline).

| **Subgroup** | **Model 1** | | **Model 2** | | **Model 3** | |
| --- | --- | --- | --- | --- | --- | --- |
|  | **HR (95% CI)** | **P^*^** | **HR (95% CI)** | **P^*^** | **HR (95% CI)** | **P^*^** |
| **All-cause mortality** | | | | | | |
| Q1 | Reference | | Reference | | Reference | |
| Q2 | 1.08(0.94-1.25) | 0.284 | 1.02(0.88-1.18) | 0.779 | 1.02(0.89-1.17) | 0.804 |
| Q3 | 1.26(1.10-1.45) | **0.001** | 1.22(1.05-1.40) | **0.007** | 1.12(0.98-1.29) | 0.101 |
| Q4 | 1.29(1.12-1.48) | **<.001** | 1.52(1.32-1.76) | **<.001** | 1.33(1.15-1.54) | **<.001** |
| **Cardiovascular mortality** | | | | | | |
| Q1 | Reference | | Reference | | Reference | |
| Q2 | 1.04(0.79-1.36) | 0.808 | 0.99(0.75-1.31) | 0.952 | 1.01(0.78-1.31) | 0.947 |
| Q3 | 1.22(0.93-1.58) | 0.149 | 1.19(0.91-1.57) | 0.200 | 1.09(0.84-1.42) | 0.503 |
| Q4 | 1.44(1.11-1.87) | **0.006** | 1.77(1.36-2.32) | **<.001** | 1.50(1.15-1.97) | **0.003** |
| **Diabetes mortality** | |  |  |  |  |  |
| Q1 | Reference |  | Reference |  | Reference |  |
| Q2 | 1.31(0.84-2.05) | 0.235 | 1.28(0.82-2.00) | 0.286 | 1.33(0.88-1.99) | 0.173 |
| Q3 | 2.17(1.44-3.27) | **<.001** | 2.18(1.43-3.30) | **<.001** | 1.91(1.30-2.81) | **0.001** |
| Q4 | 3.33(2.24-4.95) | **<.001** | 4.12(2.75-6.18) | **<.001** | 3.07(2.09-4.50) | **<.001** |

^*^P-value <0.017 was considered significant, as we had to correct our analysis for multiple testing (P-value of 0.017 was calculated as: 0.05 divided by 3).

Note: Model 1 served as the unadjusted analysis; Model 2: adjusted for age, gender, and race; Model 3: adjusted for age, gender, race, marital status, educational level, family income-poverty ratio, smoking status, alcohol use, cancer, CKD, CVD, energy intake, serum levels of TC, HDL-C, LDL-C, BUN, UA, eGFR, ALT, AST, ALB, and TBil.

Abbreviation: TyG: triglyceride-glucose; TyG-WC: TyG combining with waist circumference; Q: quartile; HR: hazard ratio; CI: confidence interval; MetS: metabolic syndrome; TC: total cholesterol; CKD: chronic kidney disease; CVD: cardiovascular disease; eGFR: estimated glomerular filtration rate; BUN: blood urea nitrogen; HDL-C: high-density lipoprotein cholesterol; LDL-C: low-density lipoprotein cholesterol; UA: uric acid; ALT: glutamic-pyruvic transaminase; AST: aspartate transaminase; ALB: albumin; TBil: total bilirubin.

**Table S18**. Sensitive analysis shows the robust association between the TyG-WHtR index with the cause and cause-specific mortality of the MetS population. (Excluding the participants with low or normal BMI at baseline).

| **Subgroup** | **Model 1** | | **Model 2** | | **Model 3** | |
| --- | --- | --- | --- | --- | --- | --- |
|  | **HR (95% CI)** | **P^*^** | **HR (95% CI)** | **P^*^** | **HR (95% CI)** | **P^*^** |
| **All-cause mortality** | | | | | | |
| Q1 | Reference | | Reference | | Reference | |
| Q2 | 1.25(1.08-1.45) | **0.004** | 1.19(1.02-1.38) | 0.024 | 1.10(0.96-1.27) | 0.160 |
| Q3 | 1.49(1.29-1.72) | **<.001** | 1.38(1.19-1.60) | **<.001** | 1.14(0.99-1.31) | 0.067 |
| Q4 | 1.54(1.34-1.78) | **<.001** | 1.80(1.56-2.09) | **<.001** | 1.45(1.25-1.68) | **<.001** |
| **Cardiovascular mortality** | | | | | | |
| Q1 | Reference | | Reference | | Reference | |
| Q2 | 1.15(0.87-1.52) | 0.311 | 1.14(0.86-1.51) | 0.352 | 1.09(0.84-1.42) | 0.508 |
| Q3 | 1.35(1.03-1.77) | 0.028 | 1.32(1.01-1.73) | 0.046 | 1.19(0.92-1.55) | 0.194 |
| Q4 | 1.60(1.23-2.09) | **<.001** | 2.05(1.56-2.68) | **<.001** | 1.69(1.29-2.23) | **<.001** |
| **Diabetes mortality** | |  |  |  |  |  |
| Q1 | Reference |  | Reference |  | Reference |  |
| Q2 | 1.16(0.74-1.81) | 0.532 | 1.13(0.72-1.78) | 0.588 | 1.39(0.92-2.11) | 0.118 |
| Q3 | 2.22(1.48-3.34) | **<.001** | 2.12(1.40-3.19) | **<.001** | 1.92(1.29-2.85) | **0.001** |
| Q4 | 3.59(2.43-5.29) | **<.001** | 4.21(2.83-6.24) | **<.001** | 3.45(2.33-5.12) | **<.001** |

^*^P-value <0.017 was considered significant, as we had to correct our analysis for multiple testing (P-value of 0.017 was calculated as: 0.05 divided by 3).

Note: Model 1 served as the unadjusted analysis; Model 2: adjusted for age, gender, and race; Model 3: adjusted for age, gender, race, marital status, educational level, family income-poverty ratio, smoking status, alcohol use, cancer, CKD, CVD, energy intake, serum levels of TC, HDL-C, LDL-C, BUN, UA, eGFR, ALT, AST, ALB, and TBil.

Abbreviation: TyG: triglyceride-glucose; TyG-WHtR: TyG combining with waist circumference to height ratio; Q: quartile; HR: hazard ratio; CI: confidence interval; MetS: metabolic syndrome; TC: total cholesterol; CKD: chronic kidney disease; CVD: cardiovascular disease; eGFR: estimated glomerular filtration rate; BUN: blood urea nitrogen; HDL-C: high-density lipoprotein cholesterol; LDL-C: low-density lipoprotein cholesterol; UA: uric acid; ALT: glutamic-pyruvic transaminase; AST: aspartate transaminase; ALB: albumin; TBil: total bilirubin.
